# Targeting host O-linked glycan biosynthesis affects Ebola virus replication efficiency and reveals differential GalNAc-T acceptor site preferences on the Ebola virus glycoprotein

**DOI:** 10.1128/jvi.00524-24

**Published:** 2024-05-17

**Authors:** Ieva Bagdonaite, Samir Abdurahman, Mattia Mirandola, Denis Pasqual, Martin Frank, Yoshiki Narimatsu, Hiren J. Joshi, Sergey Y. Vakhrushev, Cristiano Salata, Ali Mirazimi, Hans H. Wandall

**Affiliations:** 1 Department of Cellular and Molecular Medicine, Copenhagen Center for Glycomics, University of Copenhagen, Copenhagen, Denmark; 2 Public Health Agency of Sweden, Solna, Sweden; 3 Department of Molecular Medicine, University of Padua, Padua, Italy; 4 Biognos AB, Gothenburg, Sweden; 5 Department of Laboratory Medicine (LABMED), Karolinska Institute, Stockholm, Sweden; 6 National Veterinary Institute, Uppsala, Sweden; Loyola University Chicago - Health Sciences Campus, Maywood, Illinois, USA

**Keywords:** Ebola virus, GalNAc-T, mucin-like domain, O-glycosylation, tandem mass tag, mass spectrometry, glycosyltransferase, post-translational modification, viral glycoprotein

## Abstract

Ebola virus glycoprotein (EBOV GP) is one of the most heavily O-glycosylated viral glycoproteins, yet we still lack a fundamental understanding of the structure of its large O-glycosylated mucin-like domain and to what degree the host O-glycosylation capacity influences EBOV replication. Using tandem mass spectrometry, we identified 47 O-glycosites on EBOV GP and found similar glycosylation signatures on virus-like particle- and cell lysate-derived GP. Furthermore, we performed quantitative differential O-glycoproteomics on proteins produced in wild-type HEK293 cells and cell lines ablated for the three key initiators of O-linked glycosylation, GalNAc-T1, -T2, and -T3. The data show that 12 out of the 47 O-glycosylated sites were regulated, predominantly by GalNAc-T1. Using the glycoengineered cell lines for authentic EBOV propagation, we demonstrate the importance of O-linked glycan initiation and elongation for the production of viral particles and the titers of progeny virus. The mapped O-glycan positions and structures allowed to generate molecular dynamics simulations probing the largely unknown spatial arrangements of the mucin-like domain. The data highlight targeting *GALNT1* or *C1GALT1C1* as a possible way to modulate O-glycan density on EBOV GP for novel vaccine designs and tailored intervention approaches.

**IMPORTANCE:**

Ebola virus glycoprotein acquires its extensive glycan shield in the host cell, where it is decorated with N-linked glycans and mucin-type O-linked glycans. The latter is initiated by a family of polypeptide GalNAc-transferases that have different preferences for optimal peptide substrates resulting in a spectrum of both very selective and redundant substrates for each isoform. In this work, we map the exact locations of O-glycans on Ebola virus glycoprotein and identify subsets of sites preferentially initiated by one of the three key isoforms of GalNAc-Ts, demonstrating that each enzyme contributes to the glycan shield integrity. We further show that altering host O-glycosylation capacity has detrimental effects on Ebola virus replication, with both isoform-specific initiation and elongation playing a role. The combined structural and functional data highlight glycoengineered cell lines as useful tools for investigating molecular mechanisms imposed by specific glycans and for steering the immune responses in future vaccine designs.

## INTRODUCTION

Ebola virus (EBOV) is an enveloped filovirus that causes recurrent outbreaks of deadly hemorrhagic fever in Africa. Ebola virus was first discovered in 1976 in the Democratic Republic of Congo (then Zaire), and there have been at least 17 documented outbreaks with case-fatality rates varying between 39.5% and 90.9% (1). The virus spreads via person-to-person contact through cutaneous and mucosal cuts and bodily fluids. The symptoms of Ebola virus disease (EVD) vary substantially, though early symptoms often involve fever and gastrointestinal symptoms. Though the primary target cells for Ebola virus are thought to be dendritic cells and macrophages, throughout the course of infection, the virus can spread to most organs, leading to multiple organ failure, lymphocyte depletion, inhibition of innate and adaptive immunity, and, in some cases, blood vessel leakage (1). The presumed zoonotic nature of the virus poses a formidable threat in densely populated regions. The most widespread outbreaks so far were in West Africa (EBOV Makona) and The Democratic Republic of Congo in 2014 and 2018–2020, respectively, claiming numerous lives. This also led to a search of means to tame the disease and resulted in the development of therapeutic antibody cocktails and vaccines (2–6). Despite the effectiveness of ring vaccination strategies in preventing EVD, and antibody titers being detectable up to 2 years post-vaccination (7), there so far is no clinical evidence for long-term protection in humans for any of the available vaccines, and the monoclonal antibody treatment is mostly efficient in patients with lower viral load and less advanced disease (1). In addition, affordable and widely available treatments are still lacking, and identifying novel host factors modulating the course of infection is desirable.

Despite significant advances in understanding EBOV molecular biology, the impact of host glycosylation machinery on virus functionality is unclear. Glycosylation of host cell surface receptors, as well as glycosylation of EBOV glycoprotein may have regulatory roles in host-pathogen interplay, as suggested for other enveloped viruses (8–11). Glycans comprise approximately two-thirds of the EBOV GP mass, and their potential functions have mostly been probed by altering the underlying protein sequence (12–15). While targeted mutagenesis studies provide valuable information on glycosylated GP regions or predicted glycan acceptor sites that may be involved in various molecular functions, contributions of endogenous host glycosyltransferases within the infectious cycle have not been explored. Furthermore, studies tracking the progression of live EBOV infection are scarce.

**Fig 1:**
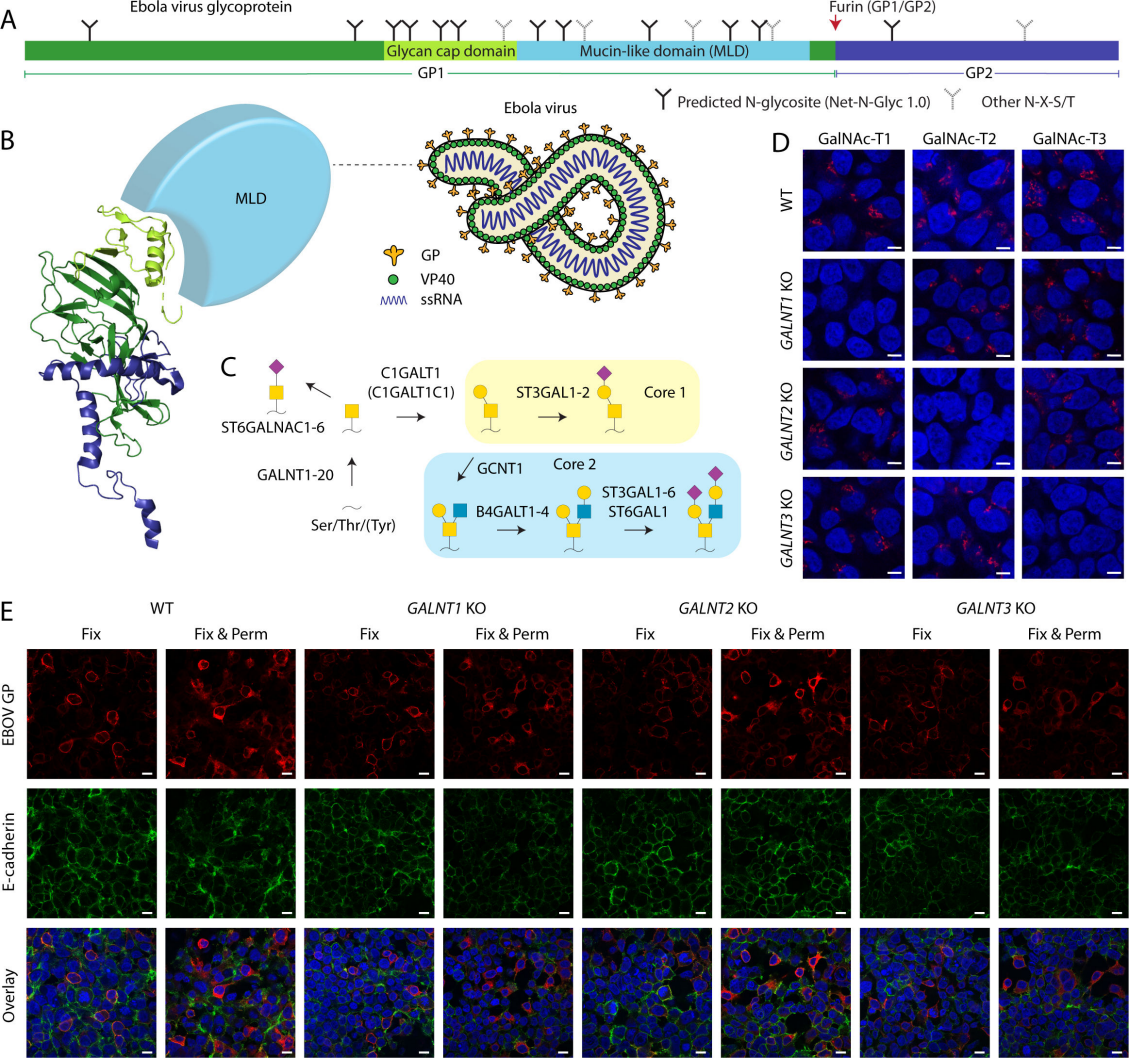
EBOV GP expression in cell lines with targeted disruptions in O-glycan biosynthesis. (**A**) Layout of Ebola virus glycoprotein. (**B**) Cartoon depiction of Ebola virus, as well as a ribbon diagram of a monomeric viral envelope glycoprotein (PDB: 6VKM). Color coding as in **A**. The mucin-like domain is not resolved and shown as a light-blue sphere. (**C**) Predominant O-glycosylation pathways in HEK293 cells. (**D**) Expression of GalNAc-T1, -T2, and -T3 in genetically engineered HEK293 cells. Scale bar, 5 µm. (**E**) Indicated cell lines transfected with a plasmid encoding full-length EBOV Makona GP were fixed with 4% PFA (Fix) 48 hours post-tranfection and co-stained for GP (red) and E-cadherin (green). Another set of cells was also permeabilized with 0.3% Triton X-100 (Fix & Perm). Scale bar, 10 µm.

The transmembrane EBOV glycoprotein (GP) is the only viral protein decorating the virion surface and is critical for mediating attachment to host cells and membrane fusion (16). Transcriptional stuttering by viral RNA polymerase creates several transcript variants of GP, of which the transmembrane glycoprotein is proteolytically processed by a furin-like host protease to yield covalently linked GP1 and GP2 that form trimers of heterodimers (Fig. 1A and B) (16, 17). GP1 mediates cellular attachment in a macropinocytosis-dependent manner by engaging TIM-1, DC-SIGN, and other C-type lectins, whereas GP2 executes fusion upon interaction with cholesterol transporter Niemann-Pick C1 protein (NPC1) that thereby acts as an endosomal entry receptor (18–24). A substantial portion of GP1 is comprised of heavily glycosylated glycan cap domain (GCD) and mucin-like domain (MLD), which collectively occlude the NPC1 binding site and therefore need to be cleaved off by cathepsins L and B for proper infectivity (Fig. 1A and B) (25–28). Furthermore, an additional priming event is needed in the endosomal compartment to ensure interaction with NPC1 and fusion (29).

Glycosylation of viral envelope glycoproteins relies on the glycosylation machinery of the host (30). The most predominant types of glycans in humans include N-linked glycans and mucin-type O-linked glycans (31). N-Linked glycans modify asparagine residues within N-X-S/T (X ≠ P) sequons and have extensively been characterized on various viral envelope glycoproteins and are easy to predict (8, 11, 32, 33). O-Glycan initiation is regulated by the competing action of 20 isoforms of polypeptide GalNAc-transferases in humans (34). While there is some knowledge on isoform-specific protein substrates, their non-redundant roles in glycosylating viral envelope proteins have not been addressed (35–38). Up to eight core structures have been described for O-GalNAc glycans, though core 1 and core 2 are the most ubiquitously encountered (Fig. 1C) (39, 40). GP1 contains 17 N-glycosylation sequons in both the GCD and the MLD; 12 of them are predicted to be utilized (Fig. 1A), and variable site usage has been reported in different expression systems (41). A few specific N-glycosites affecting EBOV conformational stability or immunogenicity have been identified and shown to provide steric shielding of host cell ligands for immune effector cells (12, 14). Furthermore, mutation of all GP1 core N-glycosites at once have been suggested to modulate interactions with host entry receptors and sensitivity to antibodies (13). The MLD is also predicted to be heavily glycosylated (42), and a number of specific O-glycosites have recently been identified on recombinant glycoproteins (41). In addition, some differences in relative abundances of O-glycan structures have been demonstrated by released glycan analysis of five Ebola virus strains, and differential glycosite patterns have been predicted by *in silico* analysis (43). However, functional studies on O-glycosylation have been limited to deletion of MLD, where it was found to be responsible for EBOV-induced cytotoxicity and vascular permeability and affect immunogenicity (12, 16, 44). The extent, regulation, and role of O-glycosylation in EBOV biology are thus still obscure. Moreover, the structure of the MLD, comprising at least half of GP’s mass (45), is poorly defined, as the region is usually omitted from crystallographic studies (Fig. 1B) (46). MLD has also been hypothesized to protect from antibody neutralization, as removal of the glycan cap during cathepsin processing would expose immunodominant residues important for receptor binding or cell fusion, which is supported by more efficient neutralization of *in vitro* pre-processed pseudotyped particles by convalescent plasma (47). Given the involvement of the MLD in various stages of viral life cycle and immune recognition, it is important to better understand its structural features, including the predominant glycan structures, their sites, and the regulation of glycan density by GalNAc-transferases.

Glycoengineering by glycogene knockout allows the introduction of subtle yet defined changes in the secretory pathway and exploring biology in a natural cellular context without introducing changes to protein sequences. Here, we took the advantage of cells in which we altered the O-glycosylation pathway to investigate functional relevance of distinct glycogenes for EBOV biology. In addition, we used tandem mass spectrometry to map O-glycosylation sites on Makona strain EBOV GP and determine the individual contribution of GalNAc-T1, -T2, and -T3 to O-glycan initiation and built a putative molecular model of the MLD structure. We furthermore discovered slower replication of EBOV in glycoengineered cell lines demonstrating that O-linked glycan initiation and elongation was necessary for optimal EBOV propagation in human epithelial cells.

## RESULTS

### EBOV GP is expressed on the surface of glycoengineered cells

Multiple studies from us and others have identified subsets of distinct glycosite specificities for individual GalNAc-T isoforms in human cells combining genetically engineered cells and mass spectrometry (35–38, 48); however, such regulated sites have not yet been reported for viral proteins. Here, we utilized a similar approach allowing to map the differentially initiated O-glycosites on an exogenously introduced viral protein. We elected to use HEK293 human epithelial cells with the genetic KO of *GALNT1*, *GALNT2*, and *GALNT3* (Fig. 1D; Fig. S1) (38) to study the potential contributions of isoform-specific glycosylation to EBOV GP. HEK293 cells are well suited for recombinant protein production and sustain propagation of live EBOV. Transfection with a construct encoding full-length EBOV GP allowed us to examine the importance of O-glycan initiation by *GALNT1*, *GALNT2*, and *GALNT3* for the surface expression of EBOV GP (Fig. 1E). In non-permeabilized cells, EBOV GP was predominantly detected on the outer rim of the cell in all cell lines, similar to endogenous surface protein E-cadherin, suggesting that alterations in O-glycosylation initiation do not significantly impair glycoprotein trafficking and verifying the suitability of the cell lines for production of recombinant glycoprotein for analysis of O-glycan sites in GP mediated by GalNAc-T1, GalNAc-T2, and GalNAc-T3.

### Different GalNAc-T isoforms glycosylate distinct sites within the MLD

**Fig 2:**
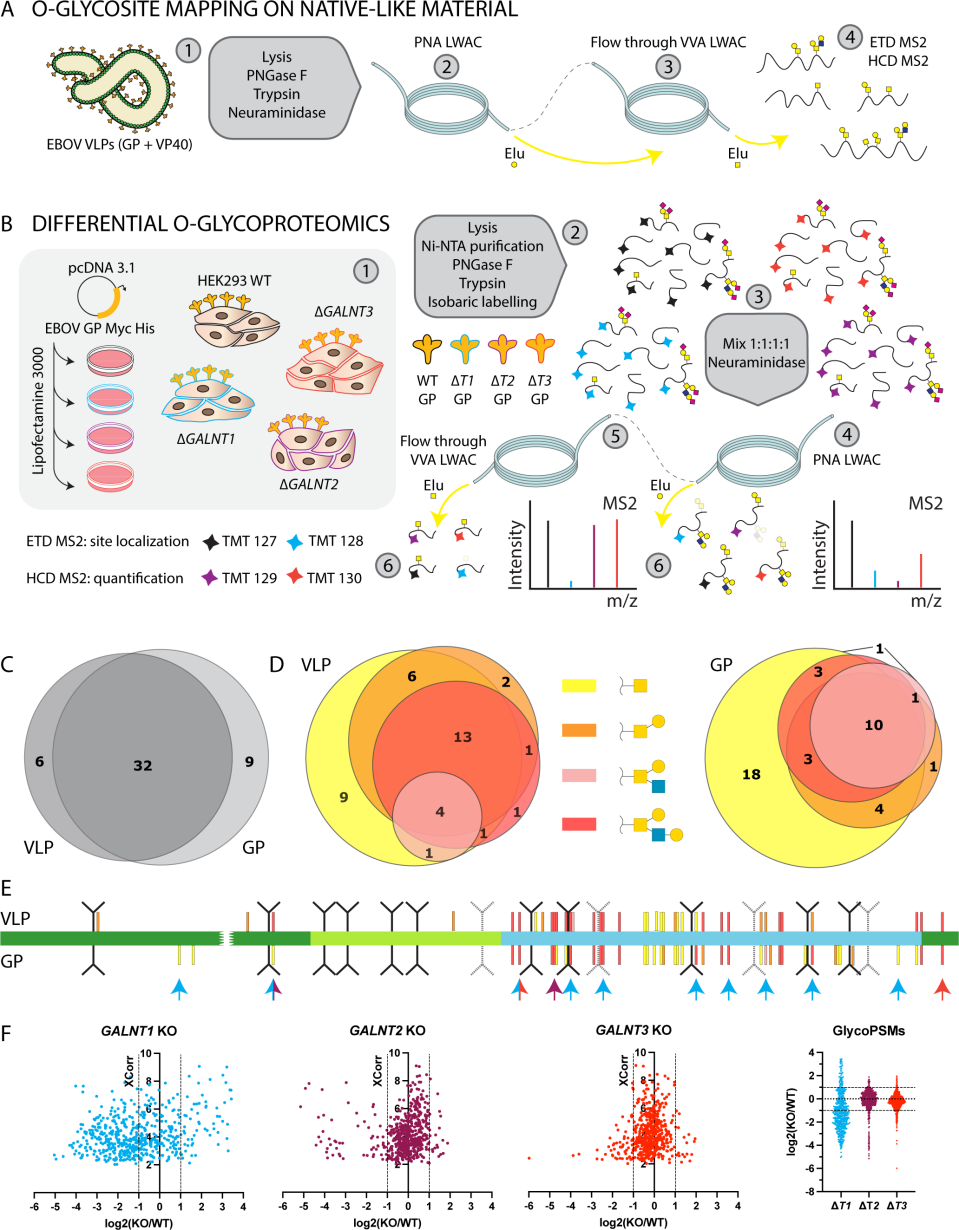
Ebola virus GP O-glycoproteomics. (**A**) Approach for mapping O-glycosites on Ebola virus-like particles comprised of GP and VP40 proteins. (**B**) Approach for mapping differentially glycosylated O-glycosites on full-length recombinant GP expressed in *GALNT* KO cell lines. (**C**) Overlap between VLP- and GP-identified O-glycosites. (**D**) The Venn diagrams were generated using DeepVenn (51) and depict the heterogeneity of identified site-specific structures, plotted separately for VLP (left) and GP (right). (**E**) Identified O-glycosites were mapped onto GP1 layout and colored based on the longest site-specific structure identified (color code as in **D**). GalNAc-T1, -T2, and -T3-regulated sites are indicated with cyan, maroon, and red arrows, respectively. (**F**) The dot plots depict distribution of TMT quantification ratios compared with wild type (log₂) for glycopeptide PSMs (peptide spectrum matches) identified in both PNA and VVA LWAC experiments plotted against XCorr (cross-correlation score) values.

**Fig 3:**
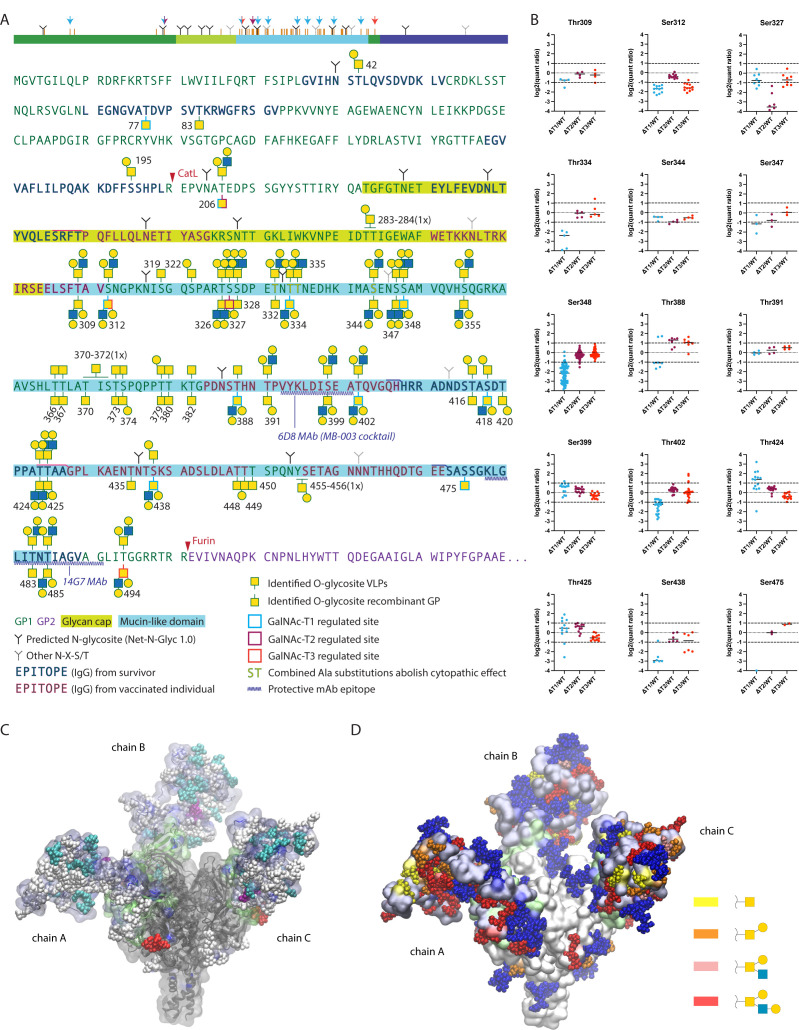
Mapping isoform-specific O-glycosites on EBOV GP. (**A**) Identified O-glycosites and most-complex unambiguously assigned site-specific structures are shown in the context of EBOV GP primary sequence, where VLP-derived sites are shown above the sequence and recombinant GP-derived sites—below the sequence. Glycosites with median quantification ratios of singly glycosylated peptides below 0.5 were considered as isoform regulated and are outlined in cyan, maroon, and red for GalNAc-T1, GalNAc-T2, and GalNAc-T3, respectively. A simplified cartoon above summarizes the data, where orange bars represent all identified O-glycosites. Color-coded arrows indicate isoform-regulated glycosites. Epitopes of protective antibodies derived from convalescent or vaccinated individuals, as well as epitopes for several protective mAbs, are annotated in the sequence (52–55). Amino acids for which combined substitutions to Ala abolish cytopathic effect are also highlighted (15). (**B**) TMT quantification ratios of single-site peptides, where each dot represents a separate PSM, and horizontal bars indicate median values. (**C**) Molecular modeling of EBOV GP with the identified O-glycans attached. The MLD was built *de novo* based on available cryo-EM/ET density maps (56, 57) of virion- and VLP-derived GP and combined with an atomic resolution structure of the GP lacking the MLD (PDB: 6HS4). The MLD is shaded in iceblue and the GCD is shaded in lime. GalNAc-T1, -T2, and -T3 regulated O-glycosites are highlighted in cyan, purple, and red, respectively. The remaining O-glycans are shown in white. Chain A contains VLP-derived glycosites, chain B contains recombinant GP-derived glycosites, and chain C contains combined maximum capacity. (**D**) Identified O-glycans are colored based on the longest site-specific structure identified, as indicated in the legend. Putative N-glycans were included in the model and are shown in blue.

We undertook a mass spectrometry-based approach to comprehensively investigate O-glycosylation on EBOV GP by identifying specific O-glycosites, their predominant structures, and site-selective initiation by GalNAc-T isoforms. In order to identify O-linked glycosylation sites on EBOV GP, we analyzed EBOV virus-like particles (VLPs) presenting the GP on native-like rod-shaped particles, generated by co-expression with VP40 protein, representing the mature glycoprotein (Fig. 2A). To determine the individual contributions of GalNAc-T1, -T2, and T3, we expressed the full-length His-tagged EBOV GP in WT and the different *GALNT* KO cell lines, purified and digested the protein, and labeled the resulting peptides with TMT isobaric mass tags allowing for relative quantification of (glyco)peptides in the different samples (Fig. 2B) (49, 50). In both approaches, we performed sequential lectin weak affinity chromatography (LWAC) of desialylated tryptic digests to enrich for abundant core 1 O-glycan structure (T, Galβ1-3GalNAcα1-O-Ser/Thr) and the biosynthetic intermediate (Tn, GalNAcα1-O-Ser/Thr) using peanut agglutinin (PNA) and *Vicia villosa* lectin (VVA), respectively. We identified 47 O-glycosites, 45 of which unambiguous, majority of which were located to MLD and glycan cap regions and were in good agreement between VLPs and purified GP, with 32 O-glycosites in common (Fig. 2C, E and 3A; Table 1; Dataset S1). PNA LWAC allowed for enrichment of not only core 1 but also core 2 structures, which were abundantly found at approximately half of the glycosites. Notably, core 2 structures could co-exist on adjacent amino acids, such as Thr326 and Ser327, Ser347 and Ser348, or Thr424 and Thr425. In contrast, we found a GP stretch, where no elongation was taking place due to dense O-glycosylation, with up to seven glycosites identified on the same peptide (366-380), predominantly as single GalNAc structures. This most likely signifies local structural differences in glycosyltransferase accessibility within different subregions of the MLD. The glycosites and the site-specific structures identified on recombinant EBOV GP were in good agreement with those identified on VLPs, representing mature glycoproteins (Fig. 2E and 3A; Table 1). The microheterogeneity observed in the total cell-purified GP, presumably resulting from capturing biosynthetic intermediates, was also seen in VLP-derived GP, suggesting a variety of proteoforms is presented on the VLP surface (Fig. 2D; Table 1). However, a larger proportion of GP-derived glycosites carried exclusively immature structures (Fig. 2D and E; Table 1).

**TABLE 1:** O-glycosites and structures

Glycosite	Recombinant GP PNA	Recombinant GP FT VVA	GalNAc-T regulation	Median KO/WT	VLPs PNA	VLPs FT VVA
Thr42					Hex1HexNAc1	HexNAc
Thr77		HexNAc	GalNAc-T1	0.43		
Thr83		HexNAc				
Ser195					Hex1HexNAc1	
Thr206		HexNAc	GalNAc-T1; GalNAc-T2	0.42; 0.27	Hex1HexNAc1, Hex2HexNAc2	HexNAc
283–284					Hex1HexNAc1	
Thr309	Hex1HexNAc1, Hex1HexNAc2, Hex2HexNAc2				Hex1HexNAc1, Hex2HexNAc2	HexNAc
Ser312	HexNAc, Hex1HexNAc1, Hex1HexNAc2, Hex2HexNAc2	HexNAc	GalNAc-T1; GalNAc-T3	0.31; 0.33	Hex1HexNAc1, Hex2HexNAc2	HexNAc
Ser319					HexNAc	
Ser322					HexNAc, Hex1HexNAc1	
Thr326	Hex1HexNAc1, Hex1HexNAc2, Hex2HexNAc2	HexNAc			HexNAc Hex1HexNAc1, Hex2HexNAc2	
Ser327	HexNAc, Hex1HexNAc1, Hex1HexNAc2, Hex2HexNAc2		GalNAc-T2	0.09	HexNAc, Hex1HexNAc1, Hex1HexNAc2, Hex2HexNAc2	HexNAc
Ser328		HexNAc			HexNAc, Hex1HexNAc1, Hex2HexNAc2	
Thr332		HexNAc			HexNAc, Hex1HexNAc1, Hex2HexNAc2	
Thr334	Hex1HexNAc1, Hex1HexNAc2, Hex2HexNAc2	HexNAc	GalNAc-T1	0.19	Hex2HexNAc2	
Thr335					Hex1HexNAc2	HexNAc
Ser344	HexNAc, Hex2HexNAc2				HexNAc, Hex1HexNAc1, Hex2HexNAc2	
Ser347	HexNAc, Hex1HexNAc1, Hex2HexNAc2				HexNAc, Hex2HexNAc2	
Ser348	HexNAc, Hex1HexNAc1, Hex1HexNAc2, Hex2HexNAc2	HexNAc, Hex1HexNAc1, Hex2HexNAc2	GalNAc-T1	0.22	HexNAc, Hex1HexNAc1, Hex2HexNAc2	HexNAc
Thr355	Hex2HexNAc2	HexNAc			HexNAc, Hex1HexNAc1, Hex2HexNAc2	
Thr366	HexNAc	HexNAc				HexNAc
Thr367	HexNAc	HexNAc			Hex1HexNAc1 (1x)	HexNAc
Thr370	HexNAc (2x)	HexNAc				HexNAc (1x)
Ser372					Hex1HexNAc1 (1x)	
Thr373		HexNAc				HexNAc
Ser374	Hex1HexNAc1	HexNAc				HexNAc
Thr379	HexNAc	HexNAc			Hex1HexNAc1	HexNAc
Thr380	HexNAc	HexNAc				HexNAc
Thr382		HexNAc				HexNAc
Thr388	Hex1HexNAc1, Hex2HexNAc2	HexNAc	GalNAc-T1	0.47	HexNAc	HexNAc
Thr391	Hex1HexNAc1	HexNAc			Hex1HexNAc1, Hex1HexNAc2, Hex2HexNAc2	HexNAc
Ser399	HexNAc, Hex1HexNAc1, Hex1HexNAc2, Hex2HexNAc2	HexNAc			HexNAc, Hex1HexNAc1, Hex1HexNAc2, Hex2HexNAc2	HexNAc
Thr402	HexNAc, Hex1HexNAc1, Hex1HexNAc2, Hex2HexNAc2	HexNAc	GalNAc-T1	0.43	HexNAc, Hex1HexNAc1, Hex1HexNAc2, Hex2HexNAc2	HexNAc
Thr416	HexNAc	HexNAc			HexNAc Hex1HexNAc1	HexNAc
Ser418	HexNAc, Hex1HexNAc2	HexNAc	GalNAc-T1	0.31	HexNAc, Hex1HexNAc1	
Thr420	Hex1HexNAc1	HexNAc				
Thr424	HexNAc, Hex1HexNAc1, Hex1HexNAc2, Hex2HexNAc2	HexNAc			Hex1HexNAc1, Hex2HexNAc2	HexNAc
Thr425	HexNAc, Hex1HexNAc1, Hex1HexNAc2, Hex2HexNAc2	HexNAc			HexNAc, Hex1HexNAc1, Hex2HexNAc2	HexNAc
Thr435		HexNAc				
Ser438	HexNAc, Hex1HexNAc1, Hex1HexNAc2, Hex2HexNAc2	HexNAc	GalNAc-T1	0.13	HexNAc, Hex1HexNAc1	HexNAc
Thr448	Hex1HexNAc1	HexNAc				
Thr449		HexNAc				
Thr450		HexNAc				
455–456 (1x)	Hex1HexNAc1					
Ser475		HexNAc	GalNAc-T1	0.06		
Thr483		HexNAc			Hex1HexNAc1, Hex2HexNAc2	
Thr485	Hex2HexNAc2	HexNAc			Hex1HexNAc1, Hex2HexNAc2	HexNAc
Thr494	Hex1HexNAc1, Hex2HexNAc2	HexNAc	GalNAc-T3	0.3	Hex1HexNAc1, Hex2HexNAc2	HexNAc

While many of the glycosites were identified on multi-glycosylated peptides, some of those sites were also covered by peptides only carrying a single O-glycan, possibly informing on the order of glycan addition. By quantifying the relative abundances of singly glycosylated peptides, we could evaluate the individual contributions of GalNAc-T1, -T2, and -T3 to specific sites (Fig. 2B, F and 3B). Based on previous work with TMT-labeled peptides, we used a twofold cutoff for identifying regulated glycosites outside of normal variation (35). Non-redundant regulated glycosites were identified for each isoform (Fig. 3A and B; Fig. S2; Table 1) demonstrating that all three GalNAc-Ts contributed to complete glycosylation of EBOV GP. We also identified non-regulated sites, suggesting either non-selective glycosylation by GalNAc-T1, -T2, or -T3 or preference by a different isoform (Fig. 3B). GalNAc-T1 had by far the largest contribution, with selective regulation of eight O-glycosites, whereas GalNAc-T2 and GalNAc-T3 were responsible for one glycosite each (Fig. 2E, F and 3A). Importantly, knockout of GalNAc-T1 had a global effect on EBOV GP glycosylation and resulted in considerable changes of both downregulated and upregulated glycopeptides (Fig. 2F), suggesting GalNAc-T1 is a major regulator of EBOV GP glycosylation. This was also supported by a notable molecular mass shift of the protein (Fig. S3). Interestingly, a big proportion of GalNAc-T1-regulated sites resided in close vicinity to N-glycosites, and most were found on de-amidated peptides, likely representing enzymatically de-N-glycosylated peptides. Importantly, these glycosites were unambiguously localized to respective serines and threonines and often harbored complex core 1 and core 2 structures, excluding the possibility of artefactual GlcNAc “stumps” at N-glycosites (Table 1; Dataset S1; Fig. S4).

To visualize the identified O-glycosites in the context of a mucin-like domain structure, we took advantage of existing cryo-EM/ET density maps of virion- (11 Å) and VLP-derived GP (56, 57), as well as an atomic resolution structure of recombinant EBOV GP lacking the MLD (PDB: 6HS4, 2.05 Å). These structures were used as a starting point to build the mucin-like domain *de novo*, while accommodating for the identified site-specific O-glycans (Fig. 3C and D). In addition, we performed a molecular dynamics simulation (100 ns) to model the behavior of the MLD, where the chain A of the trimer represents O-glycosylation of VLPs, chain B—that of recombinant GP, and chain C—maximum O-glycosylation capacity (Videos S1 to S4). The *in silico* molecular dynamic simulation provides a putative model of how O-glycans could help shape the structure of the elusive mucin-like domain (Fig. 3C and D).

### Conservation of O-glycosites across EBOV strains

**Fig 4:**
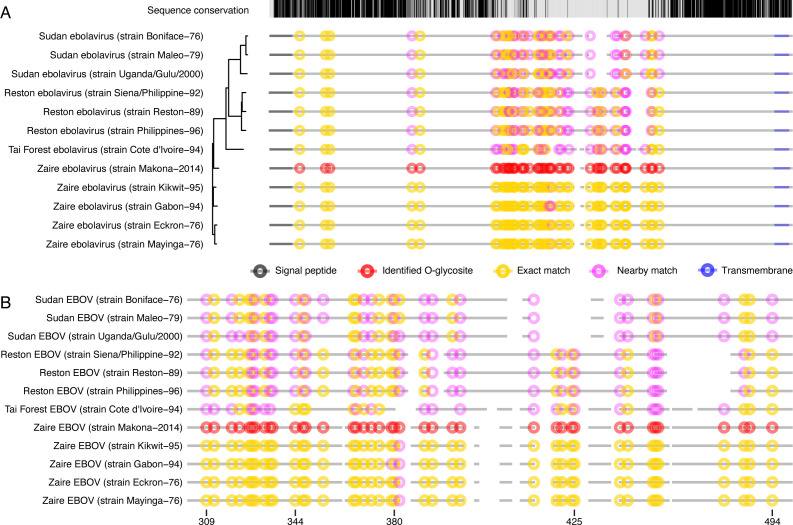
Conservation of O-glycosylated amino acids. Clustal Omega server was used to align amino acid sequences of reviewed UniProtKB entries of EBOV GP from different *Ebolavirus* strains. The sequences were ordered based on phylogenetic conservation (indicated by the phylogenetic tree). Protein backbones are depicted as broken gray lines, where spaces represent gaps in the alignment. Experimentally identified unambiguous O-glycosylation sites are marked as red circles. Conserved Ser/Thr amino acids are marked as yellow circles (including ±1 amino acids from a gap in the alignment), whereas partially conserved Ser/Thr (within ±3 amino acids from identified glycosite position) are marked as pink circles. (**A**) Full alignment. (**B**) Zoom in on the protein region containing the mucin-like domain.

To put our results in perspective, we next inspected conservation of glycosylated amino acid positions among other *Ebolavirus* strains from four species (*Tai Forest ebolavirus*, *Zaire ebolavirus*, *Reston ebolavirus*, and *Sudan ebolavirus*). We aligned the protein sequences of *Zaire ebolavirus* Makona strain with 11 other UniProtKB-reviewed GP sequences of the genus *Ebolavirus*. Given the low sequence conservation in the mucin-like domain, we marked both serines and threonines precisely matching the positions of our identified sites and those within three amino acid distance (Fig. 4A and B). Except for a few positions, we found complete conservation of glycosylated serines and threonines among the five aligned *Zaire ebolavirus* strains. Furthermore, seven positions were conserved in all 12 strains and five more positions—in all but one strain, which includes three GalNAc-T-regulated sites (Thr77, Thr206, and Ser312). Eighteen, 20, and 16 positions were conserved between Zaire and Tai Forest, Zaire and Reston, and Zaire and Sudan ebolaviruses, respectively. When taking into account nearby serines and threonines, acceptor site conservation could be identified for 34 out of the 45 unambiguous glycosites across all strains. In conclusion, we determined high conservation of glycosylated amino acid positions among Zaire ebolaviruses and substantial conservation in pairwise subtype-specific comparisons.

### O-Glycosylation of host cells modulates entry of pseudotyped VSVΔG and GP endomembrane trafficking

**Fig 5:**
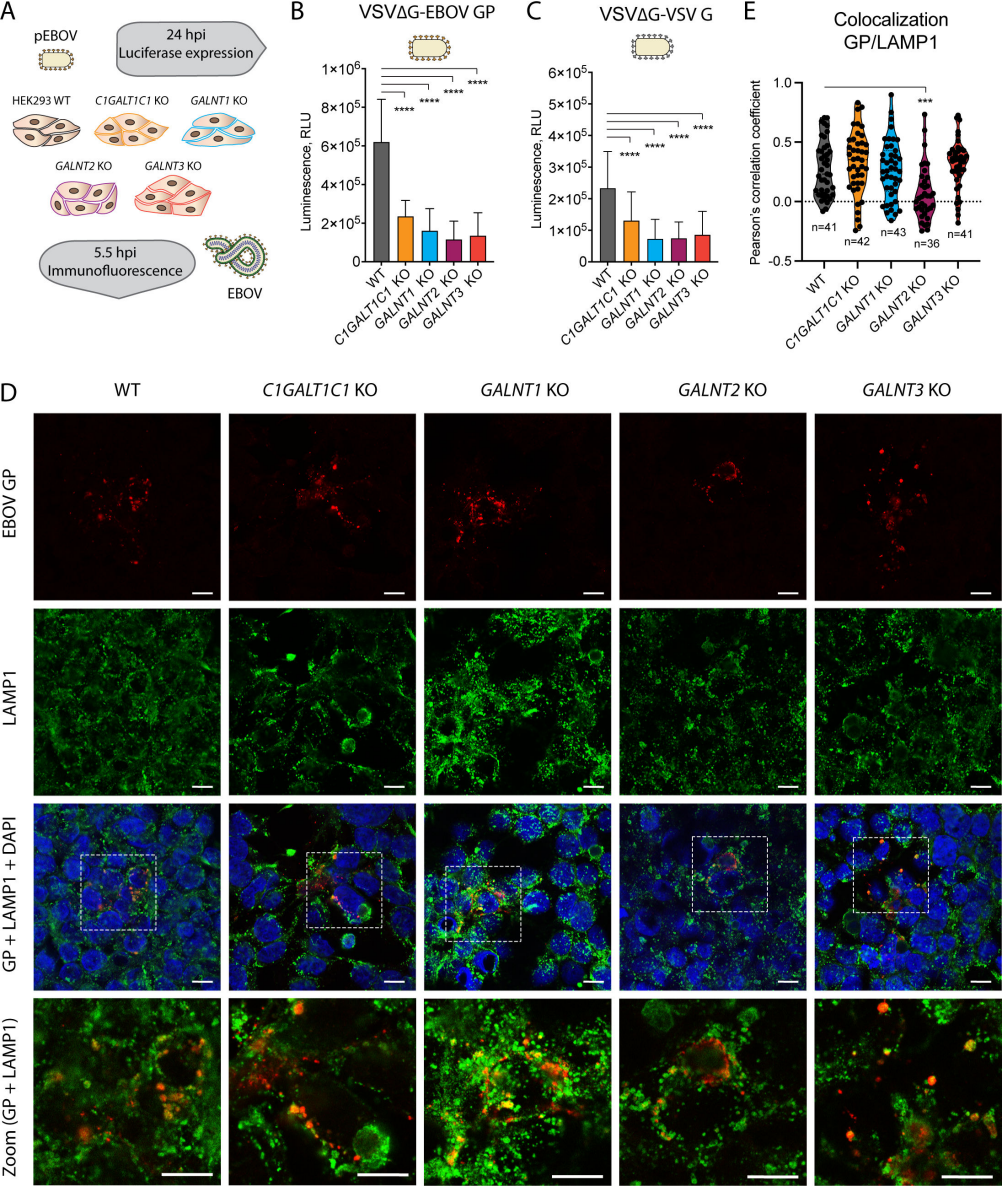
Influence of O-glycosylation on EBOV entry to cells. (**A**) Experimental setup for investigating the entry of pseudotyped and authentic EBOV. (**B**) Entry of pEBOV-VSVΔGLuc to different HEK293 KO cells shown as reporter gene expression 24 hours post-infection. The data are shown as mean + SD of five biological replicates from three independent experiments. Two-way ANOVA followed by Dunnett’s multiple comparison test was used to evaluate differences from wild type (*P < 0.05, **P < 0.01, ***P < 0.001, and **** *P* < 0.0001). (**C**) Entry of Vesicular Stomatitis Virus (VSV) G-VSVΔGLuc to different HEK293 KO cells shown as reporter gene expression 24 hours post-infection. The data are shown as mean + SD of six biological replicates from three independent experiments. Two-way ANOVA followed by Dunnett’s multiple comparison test was used to evaluate differences from wild type (*P < 0.05, **P < 0.01, ***P < 0.001, and ****P < 0.0001). (**D**) HEK293 knockout cell lines infected with the Makona isolate of Zaire EBOV at 0.01 multiplicity of infection (MOI) and fixed in acetone 5.5 hours post-infection were co-stained for EBOV GP (red) and lysosomal marker LAMP1 (green). White boxes indicate zoomed-in regions shown in bottom panels. (**E**) Up to 43 regions of interest (ROI) containing GP-positive vesicles were selected for multiple images for each cell line and colocalization estimated based on pixel-intensity correlation analysis [Pearson’s correlation coefficient (PCC)]. The violin diagrams depict the distributions of the calculated PCCs for the sampled ROIs. One-way ANOVA followed by Dunnett’s multiple comparison test was used to evaluate the differences of mean PCCs compared with wild type (*P < 0.05, **P < 0.01, ***P < 0.001, and ****P < 0.0001).

Identification of several glycosites regulated by distinct GalNAc-T isoforms, as well as non-uniform glycan density within the mucin-like domain, urged us to investigate the implications that perturbed O-glycosylation of EBOV GP or the host cell may have on basic viral functions. A successful infection relies on the uptake of viral particles into the cell and proteolytic priming of the virion-bound GP to remove the GCD and the MLD by endolysosomal proteases, followed by the fusion of host and viral membranes in acidified vesicles. To investigate the effect of host O-glycosylation on early interactions with EBOV, we performed two sets of experiments including replication-incompetent Ebola pseudovirus (pEBOV) and *bona fide* EBOV (Fig. 5A).

EBOV GP-pseudotyped recombinant vesicular stomatitis virus is lacking the gene encoding the G glycoprotein (VSVΔG-Luc) and is only able to perform a single-cycle infection, where pseudovirus entry can conveniently be traced by luciferase expression. To address GP-mediated entry to cells lacking O-glycan elongation or isoform-specific initiation, we infected the panel of glycoengineered HEK293 cells with pEBOV and quantified the luciferase reporter expression to evaluate the efficiency of infection. Diminished uptake of GP-pseudotyped VSVΔG-Luc particles was observed with all mutant cell lines (Fig. 5B). A similar effect was observed with VSV G-complemented VSVΔG-Luc (Fig. 5C) suggesting that the effect was partially accounted by GP-independent uptake possibly due to perturbations of the endocytic pathway upon manipulation of cellular O-glycans.

As the authentic EBOV utilizes distinct uptake mechanisms such as macropinocytosis, we explored possible implications on GP trafficking influenced by O-glycosylation of cellular proteins, and we looked at early stages of *bona fide* EBOV infection. To evaluate the ability of live EBOV to infect mutant cell lines, we inspected GP localization early in infection with respect to endolysosomal compartments known to play a role in EBOV infection. At 5.5 hpi, a punctate expression pattern of GP could be detected in all the five cell lines, with larger GP-positive vesicles partially colocalizing with lysosomal marker LAMP1 (Fig. 5D and E) and suggesting that EBOV was internalized and could reach the viral entry compartment. Notably, GP and LAMP1 were less likely to colocalize in *GALNT2* KO cells, whereas the two markers were more consistently colocalizing in *GALNT3* KO cells (Fig. 5E).

### EBOV exhibits diminished propagation in O-glycosylation mutant cell lines

**Fig 6:**
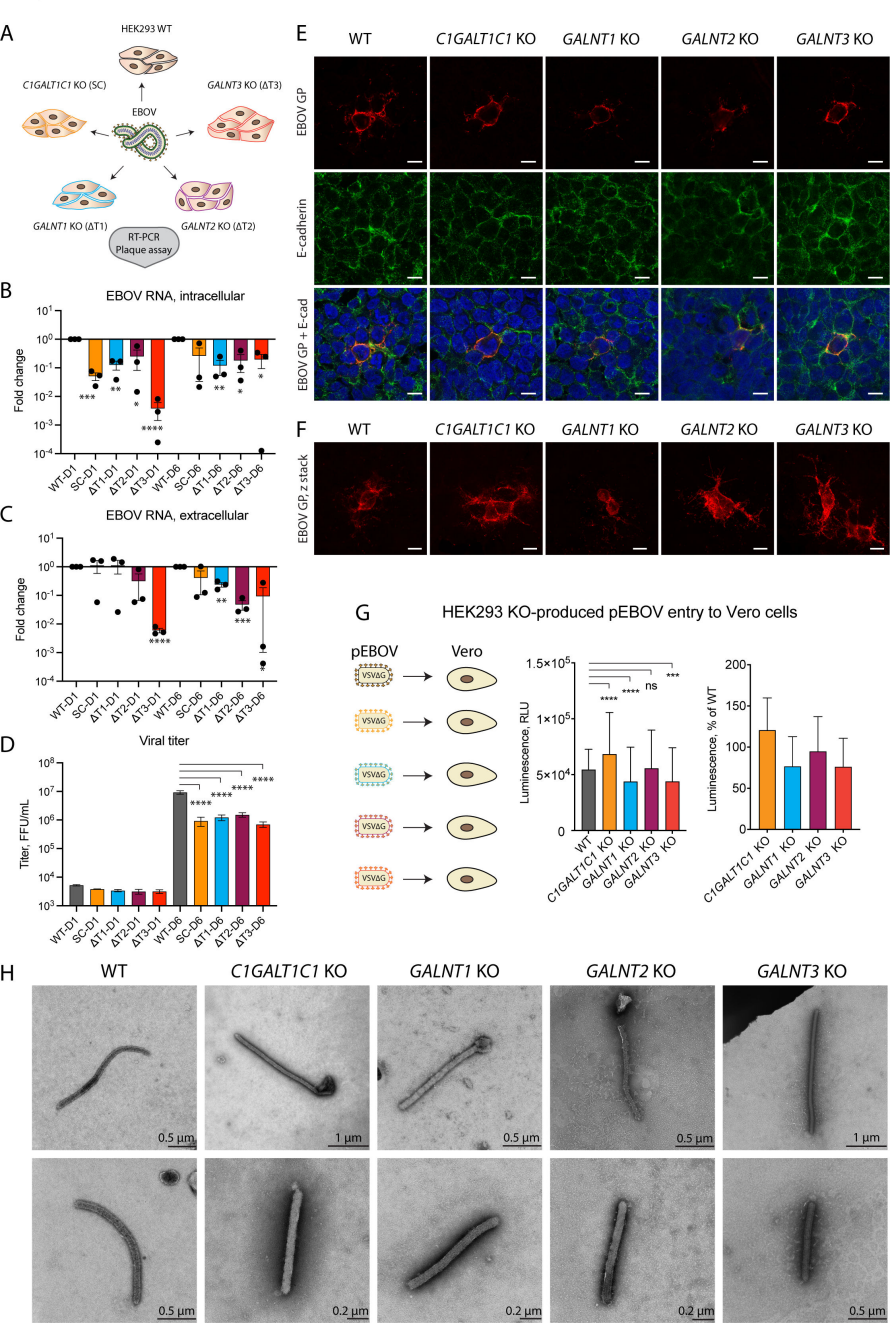
Influence of GalNAc-Ts on EBOV replication. (**A**) Experimental strategy for addressing the functional role of O-glycosylation in EBOV biology. (**B, C**) Quantitative reverse transcription PCR (RT-qPCR) analysis of viral RNA D1 and D6 post-infection of glycoengineered HEK293 cells. Expression levels are normalized to β-actin and presented as fold change compared with wild type. Data are shown as mean ± SEM of three independent experiments, where individual datapoints are also shown. One sample *t*-test was used to evaluate differences from 1 (*P < 0.05, **P < 0.01, ***P < 0.001, and ****P < 0.0001). (**B**) Intracellular RNA. (**C**) Extracellular RNA. (**D**) Plaque titration analysis of Ebola virus in cell culture supernatants of infected glycoengineered cells on D1 and D6 post-infection. Data are shown as mean ± SEM of two independent experiments. One-way ANOVA followed by Šidák’s multiple comparison test was used to evaluate differences in titers compared with wild type on the individual days post-infection (*P < 0.05, **P < 0.01, ***P < 0.001, and ****P < 0.0001). (**E, F**) HEK293 knockout cell lines infected with the Makona isolate of Zaire EBOV at 0.01 MOI and fixed in acetone 24 hours post-infection were stained for EBOV GP (red) and E-cadherin (green). (**E**) Confocal snapshots. Scale bar, 10 µm. (**F**) z-stack maximal intensity projections for EBOV GP. Scale bar, 10 µm. (**G**) Titers of HEK293 KO cell line-produced pEBOV-VSVΔGLuc harvested 14 hours post-infection and assayed on Vero cells by measuring reporter gene expression 24 hours post-infection. The data are shown as mean + SD of eight biological replicates from four independent experiments. Two-way ANOVA followed by Dunnett’s multiple comparison test was used to evaluate differences from wild type (*P < 0.05, **P < 0.01, ***P < 0.001, and ****P < 0.0001). (**H**) TEM micrographs of Ebola virions produced in the panel of glycoengineered cell lines 24 hours post-infection. Negative staining with phosphotungstic acid was used for contrasting the specimens. Scale bar is indicated on each image.

To further track the progression of EBOV infection, we imaged the suite of EBOV-infected glycoengineered cells at 24 hpi and also measured amounts of viral RNA and infectious progeny on day 1 (D1) and day 6 (D6) post-infection to capture the early and late replication stages of the virus (58), respectively (Fig. 6A). Intracellular viral replication was diminished in all mutant cell lines compared with the wild type on both day 1 and day 6 post-infection (Fig. 6B). The trend was also reflected in amounts of extracellular viral RNA on day 6 post-infection, with deletion of individual *GALNT* genes having a larger effect compared with elimination of O-glycan elongation (Fig. 6C). We then measured viral titers in cell culture supernatants to evaluate the infectivity of extracellular virions. On day 1 post-infection, viral titers in supernatants of all four knockout cell lines were comparable to the wild type (Fig. 6D). In contrast, a 6–12-fold reduction in viral titers was observed in all four knockout cell lines on day 6 post-infection (Fig. 6D). The combined data suggest the importance of both O-glycan initiation by the individual GalNAc-T isoforms and O-glycan elongation for sustained viral propagation.

To investigate whether the propagation defects were unique to Ebola virus, we performed viral propagation experiments using adenovirus 5 (AdV-5), representing a non-enveloped virus (Fig. S5).

AdV-5 replicated well in all O-glycosylation mutant cell lines on day 6 post-infection (Fig. S5A and B), though an initial delay in intracellular DNA synthesis was seen in *C1GALT1C1* KO and *GALNT1* KO on day 1 post-infection (Fig. S5A). Interestingly, compared with WT, *C1GALT1C1* KO- and *GALNT3* KO-produced AdV-5 exhibited on average 11.4-fold and 6.5-fold lower titers, respectively, on day 6 post-infection (Fig. S5C), though not statistically significant.

Immunofluorescence staining of infected wild-type cells at low MOI at 24 hpi revealed smaller and larger clusters of infected cells with predominant surface-like expression of GP (Fig. 6E and F), in contrast to the vesicular pattern observed early in infection (Fig. 5D). All KO cell lines appeared competent for GP surface expression at 24 hpi, consistent with comparable titers of progeny virus produced in all cell lines on day 1 post-infection. However, smaller foci were predominant in *GALNT2* KO cells. GP colocalized with E-cadherin on the outer rim of the cells and could also be detected along cell contacts, suggesting cell-to-cell spread and an indication that GP is presented on the cell surface (Fig. 6E). While the cumulative effect on viral titers may be influenced by efficiency of viral uptake, other factors, such as transit time through the secretory pathway, GP function, or extent of GP incorporation into viral particles, should also be considered. To investigate the ability of glycoengineered GP to mediate cellular entry, we used the different knockout cell lines to produce pEBOV (Fig. 6G). The cells were transfected with EBOV GP encoding plasmid followed by infection with VSVΔG and titration of generated stocks on Vero cells. Pseudoviruses generated in some of the mutant cell lines exhibited statistically significant, yet marginal differences compared with the wild type, where slightly lower titers were observed for *GALNT1* KO- and *GALNT3* KO-generated pEBOV, and the titer was slightly higher for *C1GALT1C1* KO-produced pEBOV (Fig. 6G). This suggests EBOV GP with altered O-glycosylation patterns is functional when presented on the VSV surface.

To investigate whether particle morphology of infectious EBOV is affected by the O-glycosylation patterns, we performed transmission electron microscopy of virions present in cell culture supernatants of infected HEK293 cells 24 hours post-infection. We could detect characteristic rod-shaped virions in supernatants of all HEK293 KO cell lines (Fig. 6H). Aside from predominantly shorter *GALNT1* KO virions, we could not detect any gross changes in particle morphology. This does not exclude potential issues with particle functionality, and ultrastructural studies would be needed to investigate GP distribution.

## DISCUSSION

Glycosylation of viral glycoproteins plays diverse important roles in infectivity and immune shielding (30). Studies on glycosylation of viral envelope proteins of highly pathogenic viruses are challenging due to several limitations, including difficulties and biosafety concerns in using the fully infectious viruses as test samples. Furthermore, due to lack of technology to eliminate specific glycosylation sites without introducing large deletions or single amino acid mutations and thereby changing the conformation of proteins, there has been a gap of knowledge regarding the effect of site-specific O-glycosylation. Here, we used a panel of genetically engineered cells in the context of natural EBOV infection to evaluate the consequences of perturbed O-glycan initiation and elongation on the cells’ ability to sustain viral propagation. In addition, we mapped the individual O-glycosites on EBOV GP and identified the GalNAc-T-regulated subsets of sites by differential O-glycoproteomics, with GalNAc-T1 having the most pronounced role.

By performing experiments with authentic EBOV and EBOV GP pseudotyped VSVΔG, we found several stages of the infectious cycle vulnerable to perturbations in the O-glycan biosynthetic pathway, including early host-pathogen interactions, and the output of infectious progeny. All investigated KO cell lines reduced the efficiency of pEBOV entry, though it was not unique to GP-mediated entry, as a similar effect was seen with VSV G-complemented VSVΔG, suggesting a broader effect on endomembrane trafficking. This was partially reflected on early interactions of *bone fide* EBOV with host cells. Early in infection, we observed some differences in the trafficking of EBOV GP—lower and enhanced GP colocalization with lysosomal marker LAMP1 was seen in G*ALNT2* KO and G*ALNT3* KO cells, respectively. Despite these initial differences, the virus was able to proceed through the infectious cycle and express GP on the cell surface at 24 hpi, though with lower RNA replication rate in the knockout cell lines. Importantly, infection with authentic Ebola virus resulted in a marked reduction of viral titers for all KO cell lines on day 6 post-infection, emphasizing the importance of cellular O-glycans for efficient propagation. The importance of O-glycans for enveloped virus biology has previously been documented (30), and we have also identified elongated O-glycans important for propagation of HSV-1 (59, 60). To test if the vulnerability to defective O-glycosylation was exclusive for enveloped viruses, we performed control experiments with adenovirus AdV-5 revealing that perturbed cellular O-glycosylation affected its propagation dynamics to a variable extent. In contrast to EBOV RNA synthesis, O-glycosylation deficiencies did not alter the DNA replication efficiency of the non-enveloped virus AdV-5 late in infection. However, a selective reduction in titers was seen in *C1GALT1C1* KO- and *GALNT3* KO-produced AdV-5. Though the surface proteins of adenoviruses do not traverse the secretory pathway, adenoviruses encode an accessory glycoprotein ADP (adenovirus death protein, E3-11.6 K), important for efficient cell lysis and release of progeny. ADP is known to be N- and O-glycosylated, and a mutant lacking O-glycosylation has been shown to exhibit a small plaque phenotype and inefficient cell lysis (61).

The lack of specific sets of glycosites on GP did not compromise its surface expression or infectivity of GP-pseudotyped VSVΔG virions or EBOV virion morphology. Nevertheless, O-glycosylation of GP has previously been proposed to modulate EBOV pathogenesis, where large deletions within the MLD, O-glycan truncation, or loss of GalNAc-T1 attenuated recombinant EBOV GP-induced cell rounding (15). Our authentic and pseudotyped EBOV data provide further insight that while trimming or reducing the number of O-glycans on GP does not necessarily impair the virus’ ability to infect cells, it may alter other direct interactions and signaling networks within host cells leading to a milder course of infection. While O-glycosylation defects did not affect GP incorporation into pseudotyped virions or their entry via clathrin-mediated endocytosis, it does not exclude detrimental effects on live virions, exhibiting entirely different morphology and entering via macropinocytosis, and is yet to be addressed. Furthermore, investigating the effect of O-glycosylation on cell types serving as primary entry points for EBOV infection *in vivo* would be advantageous.

While it is well known that EBOV GP is heavily O-glycosylated, specific glycosites or the extent of glycan density within the MLD has only recently been probed by MS methods in the present study and by Peng and colleagues (41). To address this question, we performed O-glycoproteomic analysis of EBOV VLPs to estimate the total capacity of O-glycosylation in cells expressing a complete set of GalNAc transferases and identified 38 specific O-glycosites primarily within the MLD and could confirm the majority of those sites on recombinantly expressed GP with predominant core 1 and core 2 structures. Furthermore, we found conservation of all but two O-glycan acceptor sites in four other Zaire ebolavirus strains and lower, but still substantial conservation when comparing to more distantly related Reston, Sudan, and Tai Forest ebolavirus isolates.

By performing quantitative differential O-glycoproteomic analysis using *GALNT* KO cell lines, we found that GalNAc-T1 deficiency downregulated a large proportion of rather uniformly spaced O-glycosites possibly affecting the overall glycan density on the MLD and is expected to influence its physical and immunological properties. GalNAc-T1 has previously been shown to be responsible for MLD-induced cell rounding, and compound deletion of five specific Ser/Thr amino acids has achieved the same effect (15). Four of those amino acids are homologous in our investigated strain, and we found three of them being O-glycosylated (Fig. 3A). Of those, Thr334 was selectively glycosylated by GalNAc-T1 and may be responsible for the MLD-induced cytopathic effect via a yet undefined mechanism.

Regulation of O-glycan initiation by competing GalNAc-Ts is not completely understood, and while there is no consensus sequence for O-glycosylation in general or the individual isoforms, there are slight differences in amino acid context preference by the individual catalytic domains (62, 63). Furthermore, most GalNAc-Ts are capable of long-range follow-up due to positioning by the lectin domain recognizing distant already glycosylated sites, where varying linker length between the two domains determines the range (64–66). The N-glycosite proximal GalNAc-T1-regulated sites may be a result of the lectin domain-positioned O-glycan initiation, given that GalNAc-T1 prefers to follow-up 8–10 residues N-terminally from existing O-glycosites (67, 68), which would fit well with, e.g., regulated Thr334 and Ser348 each spaced by 10 amino acids from respective Ser344 and Thr448.

We identified two O-glycosites close to a previously reported cathepsin L cleavage site (Arg200/Glu201) (25), with Thr206 regulated by GalNAc-T1 and GalNAc-T2, which could affect processing in the endolysosomal compartments. The role of O-glycosylation in regulating proteolytic cleavage is well established, and we have previously demonstrated that loss of GalNAc-T2 had a profound effect on the cellular protease network affecting processing of multiple cellular substrates (69). Similarly, we found a GalNAc-T3-regulated site (Thr494) close to the furin cleavage site (Arg501/Glu502), which may play a role in protein maturation.

In contrast to the possible regulatory capacity of isolated sites, dense O-glycosylation of the MLD is expected to contribute to immune shielding. A number of immunodominant epitopes have been identified within the MLD and the glycan cap in human survivors, in human vaccine trials, and through immunizations in animals (70–72). Interestingly, rather consistent antibody signatures have been identified in the less glycosylated GP2, whereas there is little correlation between the identified antibody epitopes in GP1 between vaccinated and convalescent individuals (52) (Fig. 3A). However, in non-human primates, vaccination approaches have generated the most immunodominant epitopes in the MLD, where class-switched antibodies were also mapped (72). This may suggest that differences in the GalNAc-transferase repertoire between the production cell lines used for vaccine generation and the repertoire in the human host cells could create an immunogenic mismatch due to differential O-glycosylation patterns (11). We found two O-glycosites (Thr483 and Thr485) in the epitope region of 14G7 neutralizing antibody shown to be protective in animal studies (53, 54, 70) (Fig. 3A). In addition, Ser399, which we find glycosylated, is part of the epitope for the 6D8 monoclonal antibody, which is included in MB-003 therapeutic antibody cocktail (53) (Fig. 3A). Notably, the 14G7 epitope-overlapping region is more immunogenic in a human survivor than in human vaccinees, whereas it is the opposite for the 6D8 epitope region (Fig. 3A) (52). Understanding these signatures in relation to GalNAc-T repertoires and glycosite occupancy would be useful for generating desirable immune responses by glycoengineering the immunogens.

Overall, our data suggest that perturbed site-specific O-glycosylation of host cells, as well as O-glycan elongation, adversely affects propagation of live EBOV and results in distinct O-glycosylation patterns on EBOV GP.

## MATERIALS AND METHODS

### Cells, plasmids and viruses

The cell lines used were HEK293T/17 (HEK293T, ATCC CRL-11268), HEK293 WT (Sigma Cat. Nr. 85120602), HEK293 *GALNT1* KO (38), HEK293 *GALNT2* KO (38), HEK293 *GALNT3* KO (38), *C1GALT1C1* KO (73) bearing truncated O-glycans (SC), VERO (VERO-ccl81, ATCC CCL-81), and Vero-E6 cells (ATCC, CRL-1586). All cell lines were maintained in Dulbecco’s modified Eagle’s medium (Life Technologies Cat. Nr. 419660), supplemented with 10% vol/vol of heat-inactivated Fetal Bovine Serum (FBS, Life Technologies Cat. Nr. 10500) and incubated at 37°C with 95% humidity and 5% CO_2_.

Zaire ebolavirus (ZEBOV isolate Ebola virus/H.sapiens-wt/SLE/2014/Makona-G3838) was propagated in Vero-E6 cells in T75 culture flasks. At day 6 post-infection, virus particles in cell culture supernatants were harvested and cleared from cell debris by centrifugation at 1,500 rpm for 10 min. The virus was then aliquoted in Eppendorf tubes, titrated in Vero-E6 cells cultured in 16-well chambered slides (Thermo Fisher Scientific) as described below, and stored at −80°C.

The plasmid expressing the Zaire ebolavirus (ZEBOV) Glycoprotein (isolate Ebola virus/H.sapiens-wt/SLE/2014/Makona-G3838), pcDNA3.1-ZEBOV-GP, was kindly provided by Stefan Pöhlmann (Deutsches Primatenzentrum GmbH, Germany). To generate the *myc*- and His-tagged version of the protein, GP sequence was subcloned to a pcDNA3.1/*myc*-His(-) plasmid. The plasmid expressing Zaire ebolavirus matrix protein VP40 fused in frame with the green fluorescent protein (GFP) (pVP40-GFP) was a gift from Christopher Basler (Mount Sinai School of Medicine, New York, USA). The plasmid expressing the VSV glycoprotein (pVSV-G) was previously described (74). The recombinant VSV encoding the luciferase in place of the VSV-G gene (VSVΔG-Luc) was provided by Michael Whitt, University of Tennessee, USA.

### EBOV infection

HEK293 wild-type and KO cells (WT, *C1GALT1C1* KO, *GALNT1* KO, *GALNT2* KO, and *GALNT3* KO) were seeded in T25 culture flasks in DMEM containing 10% FBS and 50 U/mL penicillin/streptomycin (Thermo Fisher Scientific Cat. Nr. 15070). Twenty-four hours post-seeding, cells were infected with ZEBOV at an MOI of 0.1 in infection medium (DMEM containing 2% FBS). One hour post-infection, input viruses were discarded and cells were washed once with infection medium, added fresh complete medium, and incubated further at 37°C, 5% CO_2_. At 1 and 6 days post-infection, virus particles in the culture supernatant (progeny viruses) were harvested and cleared from cell debris by centrifugation at 1,500 rpm for 10 min. Titration of progeny viruses from all HEK293 cells was done in Vero-E6 cells cultured in 16-well chambered slides (Thermo Fisher Scientific), as described below. For RNA extraction, cells and cell culture supernatants were harvested in TRIzol reagent (Thermo Fisher Scientific) at days 1 and 6 post-infection. For immunofluorescence experiments, HEK293 wild-type and knockout cells were grown on 12-well chambered glass slides and infected with ZEBOV at an MOI of 0.01 in DMEM containing 2% FBS for 1 hour at 37°C, 5% CO_2_. Thereafter, input virus was discarded and new complete medium was added to each well and incubated further at 37°C, 5% CO_2_. At 24 hours post-infection, the slides were inactivated in acetone twice, first for 10 min and then transferring to another jar with new acetone and incubating for 30 min at room temperature.

### Fluorescent focus-forming assay

Infectivity of progeny viruses produced from HEK293 wild-type and knockout cells were determined using infectious focus-forming assay. Briefly, Vero-E6 cells cultured in 16-well chambered slides were infected with 10-fold serially diluted virus for 1 hour at 37°C, 5% CO_2_. Input viruses were then discarded, and fresh complete medium was added to the cells. At 24 hours post-infection, cells were fixed in acetone for 20 min. Cells were permeabilized in phosphate buffered saline (PBS) that contained 0.1% Triton X-100 for 5 min. Slides were then incubated with rabbit anti-EBOV GP primary antibody (produced by Agrisera upon request) that was diluted in 150 mM NaCl for 1 hour, washed three times in PBS, and further incubated with FITC-conjugated goat anti-rabbit IgG (Thermo Fisher Scientific) as a secondary antibody for 30 min. All incubations were carried out at 37°C. DAPI (4',6-diamidino-2-phenylindole dihydrochloride) was used to stain cell nuclei.

### Quantitative reverse transcription PCR (RT-qPCR)

Samples from infected cells and cell culture supernatants were extracted using TRIzol reagent (Thermo Fisher Scientific). At days 1 and 6 post-infection, cell culture supernatants were harvested by mixing with TRIzol LS reagent (Thermo Fisher Scientific) at a ratio of 1 to 3. Cells in the T25 culture flask were harvested in 1 mL TRIzol reagent. Prior to RNA extraction, TRIzol-inactivated cells and cell culture supernatants were subjected to chloroform treatment, followed by RNA extraction using a PSS magLEAD 12gC machine (Precision System Science Co.) and eluted the RNA with 100 µL elution buffer. Purified samples were stored at −80°C, pending analysis.

The ZEBOV RT-PCR assay was performed in a 25-µL reaction mixture containing TaqMan Fast Virus 1-Step Master Mix (Thermo Fisher Scientific), 5 µL template RNA, 900 µM of each primer, and 200 µM of TaqMan probe. The forward primer 5′- ATGGGCTGAAAAYTGCTACAATC and reverse primer 5′-CTTTGTGMACATASCGGCAC as well the probe FAM- CTACCAGCAGCGCCAGACGGGA-TAMRA were for amplification of ZEBOV GP gene. Amplification and detection of the vRNA were performed in a StepOne Plus Real-Time PCR Machine (Applied Biosystems). The cycling conditions were as follows: 50°C for 5 min and 45 cycles of 95°C for 20 sec and 95°C for 3 sec and 60°C for 30 sec. Human beta-actin was used as an endogenous gene control to confirm the integrity of the extraction reagent and RNA recovery as well as to normalize the levels of intracellular viral RNAs.

### Ebola virus-like particle production

Zaire ebolavirus-Like particles (EBOVLPs) were produced by transfection of HEK293T cells with a plasmid expressing the EBOV matrix protein VP40 fused to GFP (VP40-GFP) along with a construct expressing either the ZEBOV GP—strain Makona—or the VSV envelope G glycoprotein. Cells seeded in 100-mm plates were transfected with 11.5 µg of pVP40-GFP and along with 0.5 µg of pcDNA3.1-ZEBOV-GP or pVSV-G by calcium-phosphate (75). Cell culture supernatants were collected 48 hours after transfection, and cell debris were cleared by centrifugation (1,200 rpm for 7 min at 4°C). EBOVLP production was confirmed by cytofluorimetric analyses of transduced Vero CCL81 cells as previously described (76). Next, VLPs were purified and concentrated by two ultracentrifugation steps at 27,000 rpm for 2 hours in 20% sucrose cushion, then aliquoted, and stored at −80°C.

### Pseudotyped virus production and titration

ZEBOV GP pseudotyped VSVΔG-Luc (GP-pseudotyped virus) was generated as previously described (77). Briefly, HEK293T cells were transfected by calcium-phosphate protocol with 16 µg of pcDNA3.1-ZEBOV-GP plasmid and, 24 hours later, infected with the recombinant VSVΔG-Luc virus at the MOI of 4 fluorescent focus-forming units (FFU)/cell. After 24 hours, the pseudotyped virus was harvested and stored at −80°C. Viral titer was evaluated as FFU/mL using the anti-VSV-Matrix protein 23H12 antibody (Kerafast Inc.) to detect infected cells.

### EBOV-pseudotype (pEBOV_KO) production in HEK293 knockout cell lines

HEK293 were seeded in poly-Lysine-coated six-well plates and transfected with the pcDNA3.1-ZEBOV-GP plasmid as described above. Six hours later, cells were washed and fresh medium was added. The day after, cells were infected for 1 hour with the VSVΔG-Luc virus at MOI 4 FFU/cell, washed twice in PBS, and incubated in complete medium for 14 hours. Next, medium was recovered and centrifuged at 3,500 rpm at 4°C for 6 min to remove cellular debris. Then, aliquots were made and stored at −80°C for further use. The viral progeny production was evaluated infecting semi-confluent Vero-CCL-81 cells seeded in 96-well plates. After 1 hour, cells were washed in PBS and cultured for additional 24 hours before the evaluation of the Luc expression using the Steady-Glo Luciferase Reagent (Promega), which was added to the cells for 15 min. The luminescence was detected with a VICTOR Multilabel Plate Reader (PerkinElmer) as relative-luminescence unit (normalized on a scale factor of 1 s).

### pEBOV infection of HEK293 cells

One day before the assay, 2 × 10^4^ HEK293 wild-type and knockout cells were seeded in poly-Lysine-coated 96-well plates. The day after, cells were washed with PBS and infected with pEBOV at MOI 0.01 for 1 hour. Then the cells were washed with PBS and new complete medium was added. After 24 hours, the reporter gene expression was detected as described above, to evaluate the efficiency of infection.

### AdV-5 infection of HEK293 cells

Infection of HEK293 wild-type and KO cells with Human Adenovirus-5 (AdV-5, ATCC CCL-2) was done essentially as described with EBOV infection. Briefly, cells were seeded on six-well plates overnight. The cells were then infected the next day with AdV-5 at an MOI of 0.01 for 1 hour at 37°C, 5% CO_2_. Input viruses were discarded, and fresh complete medium containing 5% FBS and penicillin/streptomycin (Thermo Fisher Scientific) was added to the cells. At days 1 and 6 post-infection, cells and cell culture supernatants were harvested. Cell culture supernatants were cleared of cell debris by centrifugation at 626 × *g* for 5  min and used for studying the kinetics of the viral progeny propagation in Vero-E6 cells, as described below. The level of the virus in the culture supernatant was also determined by qPCR. The monolayer cells were washed one time in PBS and harvested in 380 µL of nuclisens easymag lysing buffer, before both cells and supernatants were subjected to viral DNA isolation.

### Replication kinetics of AdV-5 produced in HEK293 wild-type and KO cells

Equal amounts of progeny viruses from HEK293 wild-type and knockout (KO) cell culture supernatants were 10-fold serially diluted in DMEM with 5% FBS and used to infect Vero-E6 cells in 96-well plates for 6 days at 37°C, 5% CO_2_. At days 1 and 6 post-infection, the change in cell morphology associated to the cytopathic effect of the virus was monitored over time a under microscope. The results were recorded as positive or negative wells, and the titers were calculated by the Spearman-Karber formula.

### Isolation and purifying adenoviral DNA

For adenoviral DNA extraction, cells and cell culture supernatants of infected HEK293 cells were harvested in nuclisens easymag lysing buffer (Biomérieux) at days 1 and 6 post-infection. Three hundred seventy microliters of cells and cell culture supernatant lysates of each were mixed with 30 µL of Proteinase-K and incubated at 56°C until complete lysis was achieved, vortexing three to four times during the incubation period. After a complete lysis, nucleic acid extraction was done using a PSS magLEAD 12gC extraction machine (Precision System Science Co.) according to the extraction kit protocol and the DNA was eluted with 50 µL elution buffer. Purified samples were stored at −80°C, pending analysis.

### qPCR

Nucleic acids were extracted from 370-µL aliquots of cells and cell culture supernatant lysates and eluted to 50 µL. Five microliters of the elution was used for each reaction. Adenoviral DNAs were amplified using the L2 primer and probe set as described elsewhere (78). Briefly, the primer pairs bind to the L2 gene of the adenovirus serotype 5 and amplify a 61-bp fragment. Amplification of the adenoviral DNAs were done using TaqMan universal PCR Master Mix Kit (Thermo Fisher scientific), and the reaction conditions were as follows: 10 min at 95°C followed by 45 cycles of 10 s at 95°C and 30 s at 60°C. Each sample was analyzed in duplicate together with no template and AdV-5-positive control.

### Transfection

For immunofluorescence experiments, the panel of HEK293 cells grown on poly-Lysine (Sigma-Aldrich Cat. Nr. P6282) pre-coated [2.5 µg/mL in MQ H_2_O 30 min at room temperature (RT)] glass cover slips in 24-wells was transfected with 0.5 µg/well of pcDNA3.1/*myc*-His(-) plasmid encoding *myc*- and His-tagged full-length EBOV GP (Zaire ebolavirus isolate Ebola virus/H.sapiens-wt/SLE/2014/Makona-G3838) using Lipofectamine 3000 (Thermo Fisher Scientific Cat. Nr. L3000) according to the manufacturer’s instructions. Non-transfected cells were used as controls. At 48 hours post-transfection, the cells were washed 3× with Hanks′ Balanced Salt Solution (HBSS, Sigma-Aldrich Cat. Nr. H8264) and fixed with 4% PFA in PBS (Ampliqon Cat. Nr. AMPQ44154.1000) for 10 min at RT followed by 3× more washes. If needed, the cells were permeabilized with 0.3% Triton X-100 in HBSS for 3 min followed by 3× washes with PBS and blocked with 2.5% BSA in PBS and 0.03% sodium azide at 4°C until staining. For western blotting experiments, the panel of HEK293 cells grown in six-wells was transfected with 2 µg/well of the plasmid and harvested at 48 hours post-transfection. The cells were washed with PBS and lysed in modified RIPA buffer (50 mM Tris pH 7.5, 150 mM NaCl, 1% NP-40, 0.1% Na deoxycholate, and 1 mM EDTA) supplemented with protease inhibitor cocktail (cOmplete, EDTA-free, Roche Cat. Nr. 11873580001) and phosphatase inhibitors for 30 min at 4°C with agitation, followed by sonication using a sonic probe on ice. The lysates were centrifuged at 10,000 × *g* for 10 min at 4°C, and the supernatants were used for subsequent western blotting. For protein purification, HEK293 WT, *GALNT1* KO, *GALNT2* KO, and *GALNT3* KO cells were grown in 3× P150 dishes each and transfected with 23 µg/dish of the plasmid and harvested at 48 hours post-transfection. The plates were chilled on ice and washed with ice-cold PBS twice, and cells were gently scraped in ice-cold PBS followed by centrifugation at 500 × *g* for 10 min at 4°C. After decanting most of PBS, pellets were dislodged and pooled for each cell type, followed by centrifugation at 800 × *g* for 10 min at 4°C. PBS was removed, and pellets were stored at −80°C.

### EBOV GP purification

Cell pellets were thawed on ice in 0.1% RapiGest (Waters Cat. Nr. 186001861) in Equilibration buffer (25 mM Tris-HCl 300 mM NaCl pH 7.5) supplemented with protease inhibitor cocktail (cOmplete, EDTA-free, Roche Cat. Nr. 11873580001) followed by sonication using a sonic probe on ice. His-tagged proteins were purified using HisPur Ni-NTA resin (Thermo Fisher Scientific Cat. Nr. 88222) according to the manufacturer’s instructions (batch protocol). Briefly, the lysates were cleared by centrifugation at 10,000 × *g* for 10 min at 4°C and applied to pre-equilibrated HisPur Ni-NTA resin followed by a 1-hour incubation on an end-over-end rotator. The resin was washed 3 × 5 min with wash buffer (25 mM Tris-HCl 300 mM NaCl pH 7.5, 10 mM imidazole), and bound proteins were eluted in elution buffer (25 mM Tris-HCl 300 mM NaCl pH 7.5, 250 mM imidazole). Protein concentration was measured using Pierce 660 nm Protein Assay Kit (Thermo Fisher Scientific Cat. Nr. 22662) and 400 µg of each cell type-derived protein taken for subsequent MS sample preparation.

### Immunofluorescence

Fixed cover slips or teflon-coated slides were incubated with primary antibodies diluted in 2.5% BSA (Sigma-Aldrich Cat. Nr. A3294) in PBS and 0.03% sodium azide over night at 4°C or RT for 1 hour: rabbit anti-EBOV GP (1:300, produced by Agrisera upon request), mouse anti-LAMP1 (1:200, SCBT Cat. Nr. Sc-20011), goat anti-E-cadherin (1:200, R&D Systems Cat. Nr. AF648), rabbit anti-Myc (1:200 Abcam Cat. Nr. ab152146), or undiluted hybridoma supernatants [mouse anti-GalNAc-T1 (4D8), mouse anti-GalNAc-T2 (4C4), mouse anti-GalNAc-T3 (2D10)], followed by 3× washes with PBS and 1-hour incubation at RT with secondary antibodies: donkey anti-rabbit IgG AF546 (1:500), donkey anti-rabbit IgG AF594 (1:500), donkey anti-goat IgG AF488 (1:500), goat anti-rabbit IgG AF488 (1:500), goat anti-mouse IgG AF594 (1:500), and goat anti-mouse IgG AF488 (1:500), all from Thermo Fisher Scientific. After 3× washes with PBS, cover slips were incubated with 0.1 µg/mL DAPI solution for 4 min followed by 3× washing and mounting with Prolong Gold Antifade Reagent (Thermo Fisher Scientific Cat. Nr. P36930). Teflon-coated slides were mounted using Prolong Gold Antifade Reagent with DAPI (Thermo Fisher Scientific Cat. Nr. P36935). Fluorescence micrographs and z-stacks were obtained on a Zeiss LSM710 confocal microscope. Images were assembled using Adobe Photoshop, Adobe Illustrator, or Zeiss ZEN Lite software.

### Western blotting

Thirty micrograms of protein extracts was divided into two aliquots, one of which was treated with 2 U of PNGase F (Roche Cat. Nr. 11365177001) at 37°C for 1 hour. Samples were then mixed with 4× NuPAGE sample buffer (Thermo Fisher Scientific Cat. Nr. B007) and 10 mM dithiothreitol (DTT), heat denatured (95°C 5 min), and separated on Novex 4%–12% gradient gel (Bis-Tris) (Thermo Fisher Scientific Cat. Nr. NP0329) in 1× NuPAGE MES running buffer (Thermo Fisher Scientific Cat. Nr. NP0002), followed by transfer onto the nitrocellulose membrane in 20% MeOH in running buffer at 320 mA for 1 hour. Membranes were blocked with 5% skim milk in TBS-T and blotted with rabbit anti-EBOV GP (1:800, produced by Agrisera upon request) antibody overnight at 4°C, followed by goat anti-rabbit Igs-HRP (1:4,000, DAKO Cat. Nr. P0448) for 1 hour at RT. Membranes were developed using the Pierce ECL Kit (Thermo Scientific) and visualized using the ImageQuant LAS4000 System.

### Transmission electron microscopy

Cell culture supernatants from HEK293 WT and different KO cell lines harvested at 24 hours post-infection were inactivated by mixing 1:1 with 2.5% freshly prepared glutaraldehyde and incubating for 1 hour at room temperature. Samples were applied to copper grids (Gilder Grids, G400) and negative stained with phosphotungstic acid. Grids were analyzed using a CM100 transmission electron microscope (FEI/Philips) equipped with TWIN objective lens and a side-mounted Olympus Veleta camera with a resolution of 2,048 × 2,048 pixels (2K × 2K). Images were recorded using ITEM software.

### O-Glycoproteomic sample preparation

For differential O-glycoproteomic analysis, 400 µg of each cell type-derived protein in equal volumes of elution buffer was diluted to 1 mL using 0.5% RapiGest in 250 mM AmBic (ammonium hydrocarbonate) and H_2_O resulting in a final concentration of 0.1% RapiGest and 50 mM AmBic. Proteins were reduced by adding up to 5 mM DTT (Sigma-Aldrich Cat. Nr. D0632) and incubating at 60°C for 45 min followed by alkylation with 10 mM iodoacetamide (IAA, Sigma-Aldrich Cat. Nr. I1149) at RT for 30 min in darkness. The proteins were then treated with 8 U PNGase F (Roche Cat. Nr. 11365177001) at 37°C for 3 hours followed by 19 µg/sample of trypsin (Roche Cat. Nr. 11418025001) at 37°C for 13 hours. Digests were acidified with trifluoracetic acid and peptides purified using Sep-Pak (1cc) C18 cartridges (Waters Cat. Nr. WAT023590) [1× CV MeOH; 1× CV 50% MeOH 0.1% FA; 3× CV 0.1% TFA; load twice; 3× CV 0.1% FA; and elute in 2× CV of 50% MeOH 0.1% FA (CV = column vol)]. Peptide concentrations were measured using NanoDrop A205. Two hundred micrograms of peptides from each sample was taken and most of the solvent was evaporated using SpeedVac and then up to 1 mL H_2_O was added and freeze dried. Dried peptides were reconstituted in 100 µL TEAB and labeled with TMTsixplex Reagents (TMT 127, TMT 128, TMT 129, and TMT 130, Thermo Fisher Scientific Cat. Nr. 90061) according to the manufacturer’s instructions. One percent of each reaction was mixed, dried, and submitted to liquid chromatography–mass spectrometry (LC-MS) analysis for a ratio check. After confirming equal ratios, all four channels were mixed and dried with SpeedVac. The peptide mix was then reconstituted in 50 mM sodium acetate pH 5 and treated with 0.25 U/mL *Clostridium perfringens* neuraminidase (Sigma-Aldrich Cat. Nr. N3001) at 37°C for 3 hours. For O-glycoproteomic analysis of EBOV VLPs, 300 µL of VLPs was diluted with 0.1% RapiGest in 50 mM AmBic up to 1.3 mL, then sonicated using a sonic probe on ice, and heated at 80°C for 10 min, and the protein concentration was measured using the Pierce BCA Assay Kit. Approximately 800 µg of VLP lysate was then reduced and alkylated as described above and treated with 5 U PNGase F (Roche Cat. Nr. 11365177001) at 37°C for 12 hours followed by 12 hours with 11 µg of trypsin. PNGase F treatment was then repeated followed by a 2-hour incubation with 4 µg of trypsin. Peptides were purified as described above, concentrated by SpeedVac, and treated with 0.15 U/mL neuraminidase at 37°C for 3 hours.

### LWAC enrichment

Sequential PNA and VVA LWAC enrichment was performed as previously described (60). Elution fractions were desalted using self-made Stage Tips (C18 sorbent from Empore 3M) and submitted to LC-MS and higher-energy collisional dissociation/electron-transfer dissociation tandem mass spectrometry (HCD/ETD-MS/MS).

### Mass spectrometry analysis

Liquid chromatography–tandem mass spectrometry (LC-MS/MS) site-specific O-glycopeptide analysis was performed using EASY-nLC1200 ultra-high-performance liquid chromatography (UHPLC) (Thermo Fisher Scientific) interfaced via a nanoSpray Flex Ion Source to an Orbitrap Fusion Lumos Tribrid MS (Thermo Fisher Scientific) or an on EASY-nLC1000 UHPLC (Thermo Fisher Scientific) interfaced via a PicoView nanoSpray Ion Source (New Objectives) to Orbitrap Fusion Mass Spectrometer (Thermo Fisher Scientific). The nLC was operated in a single analytical column set up using PicoFrit Emitters (New Objectives, 75 mm inner diameter) packed in house with Reprosil-Pure-AQ C18 phase (Dr. Maisch, 1.9 mm particle size, 19–21 cm column length). Each sample was injected onto the column and eluted in gradients from 3% to 32% B in 95 min, from 32% to 100% B in 10 min, and 100% B in 15 min at 200 nL/min [Solvent A, 100% H_2_O; Solvent B, 80% acetonitrile; and both containing 0.1% (vol/vol) formic acid, EASY nLC-1200] and from 3% to 25% B in 95 min, from 25% to 80% B in 10 min, and 80% B in 15 min at 200 nL/min [Solvent A, 100% H_2_O; Solvent B, 100% acetonitrile; both containing 0.1% (vol/vol) formic acid, EASY nLC-1000].

A precursor MS1 scan (*m*/*z* 350–1,700) was acquired in the Orbitrap at the nominal resolution setting of 120,000, followed by Orbitrap HCD-MS2 and ETD-MS2 at the nominal resolution setting of 50,000 of the five most abundant multiply charged precursors in the MS1 spectrum; a minimum MS1 signal threshold of 50,000 was used for triggering data-dependent fragmentation events. For EBOVLP samples, stepped collision energy ±5% at 27% was used for HCD MS/MS fragmentation and the charge-dependent calibrated ETD reaction time was used with CID supplemental activation at 30% collision energy for ETD MS/MS fragmentation. For TMT-labeled samples, stepped collision energy ±5% at 45% was used for HCD MS/MS fragmentation and the charge-dependent calibrated ETD reaction time was used with CID supplemental activation at 30% collision energy for ETD MS/MS fragmentation.

For the site-specific glycopeptide identification, the corresponding HCD MS/MS and ETD MS/MS data were analyzed by Proteome Discoverer 1.4 Software (Thermo Fisher Scientific) for EBOVLP samples or Proteome Discoverer 2.2 Software (Thermo Fisher Scientific) for EBOV GP samples using Sequest HT as a searching engine. Carbamidomethylation at cysteine, as well as TMT at the N-terminus and lysine (TMT-labeled samples only), weas used as fixed modifications, and oxidation at methionine, asparagine deamidation, and HexNAc, Hex1HexNAc1, Hex1HexNAc2, and Hex2HexNAc2 at serine/threonine/tyrosine were used as variable modifications. Precursor mass tolerance was set to 10 ppm, and fragment ion mass tolerance was set to 0.02 Da. TMT reporter ions from HCD MS2 data were used for quantification. For peak integration, the integration tolerance window was set to 20 ppm and the integration method was chosen as the most confident centroid. For calculation of TMT ratios, the respective *GALNT* KO channels were used as the numerator and the WT channel was selected as the denominator. PSMs with TMT ratios below 0.5 were considered as downregulated. Data were searched against the human-specific UniProt KB/SwissProt-reviewed database downloaded in January 2013 and construct-dependent viral protein sequence databases. Processed data were filtered to include PSMs with “High” Confidence, Search Engine Rank “1,” and XCorr value of at least 1.2. All spectra of interest were manually inspected and validated to prove the correct peptide identification and glycosite localization.

### Molecular modeling

The initial three-dimensional model of the fully glycosylated Ebola virus GP was built using the graphical interface of YASARA (79). The model was based on the crystal structure of Ebola GP (PDB entry 6hs4, resolution 2.05 Å), which lacks most of the residues in the range 294–501. This amino acid sequence—which includes the MLD—was built *de novo*, and the N-glycans and O-glycans (taken from an in-house 3D library) were attached to the protein based on information shown in Fig. 3. The glycopeptide was relaxed by MD simulation and then subsequently fitted into the crystal structure, taking into account partly resolved parts (residues 302–310 and 471–478) and guided by available cryo-EM/ET density maps of virion- and VLP-derived GP (56, 57). The simulation was performed with GP1 still attached to GP2. The other missing loops in PDB entry 6hs4 were also subsequently modeled, and N- and O-glycans were attached. Single amino acid mutations were introduced in order to match the target sequence (isolate Ebola virus/H.sapiens-wt/SLE/2014/Makona-G3838). The trimeric 3D structure was finally adjusted so that chain A contains VLP-derived glycosites, chain B—recombinant GP-derived glycosites, and chain C—combined maximum capacity. Since each chain contained up to 46 O-glycans and 17 N-glycosylation sites, it was instrumental to cross-check the glycosylation pattern in the molecular system during the building process and prior to simulation using Conformational Analysis Tools (CAT).

Finally, the trimeric glycoprotein was solvated in 0.9% NaCl solution (0.15 M) and simulations were performed at 310 K using the AMBER14 force field (80–82). The box size (approx. 185Åx185Åx185Å, 643911 atoms) was rescaled dynamically to maintain a water density of 0.996 g/mL. Simulations were performed at a rate of 4 ns/day using YASARA with GPU acceleration in “fast mode” (4 fs time step) (83) on “standard computing boxes” equipped, e.g., with one 12-core i9 CPU and NVIDIA GeForce GTX 1080 Ti.

CAT (http://www.md-simulations.de/CAT/) was used for the analysis of trajectory data, general data processing, and generation of scientific plots. VMD (84) was used to generate molecular graphics.

## Data Availability

The mass spectrometry proteomics data have been deposited to the ProteomeXchange Consortium via the PRIDE (85) partner repository with the data set identifier PXD036213. All other data are included in the paper.

## SUPPLEMENTAL MATERIAL

Dataset S1 jvi.00524-24-s0001.xlsx Identified O-glycopeptides.

Figure S1 jvi.00524-24-s0002.jpg GalNAc-T expression in HEK293 KO cell lines.

Figure S2 jvi.00524-24-s0003.jpg TMT reporter ion abundances of monoglycosylated peptides.

Figure S3 jvi.00524-24-s0004.jpg EBOV GP expression in HEK293 WT and KO cell lines.

Figure S4 jvi.00524-24-s0005.jpg Examples of O-glycosites localized close to/within N-glycan sequons carrying Hex2HexNAc2 modifications.

Figure S5 jvi.00524-24-s0006.jpg AdV-5 replication in glycoengineered cell lines.

Supplemental text jvi.00524-24-s0007.docx Legends for supplemental videos.

Video S1 jvi.00524-24-s0008.mov MD simulation of O-glycosylated EBOV GP with isoform-specific glycosites highlighted.

Video S2 jvi.00524-24-s0009.mov 3D model of O-glycosylated EBOV GP with isoform-specific glycosites highlighted.

Video S3 jvi.00524-24-s0010.mov MD simulation of O-glycosylated EBOV GP with O-glycans colored by structure.

Video S4 jvi.00524-24-s0011.mov 3D model of O-glycosylated EBOV GP with O-glycans colored by structure.

ASM does not own the copyrights to Supplemental Material that may be linked to, or accessed through, an article. The authors have granted ASM a non-exclusive, world-wide license to publish the Supplemental Material files. Please contact the corresponding author directly for reuse.
