# Supplementary figures and images for: Targeting host O-linked glycan biosynthesis affects Ebola virus replication efficiency and reveals differential GalNAc-T acceptor site preferences on the Ebola virus glycoprotein
Source: J Virol. 2024 May 17;98(6):e00524-24. doi: 10.1128/jvi.00524-24 (PMC11237518; doi:10.1128/jvi.00524-24)

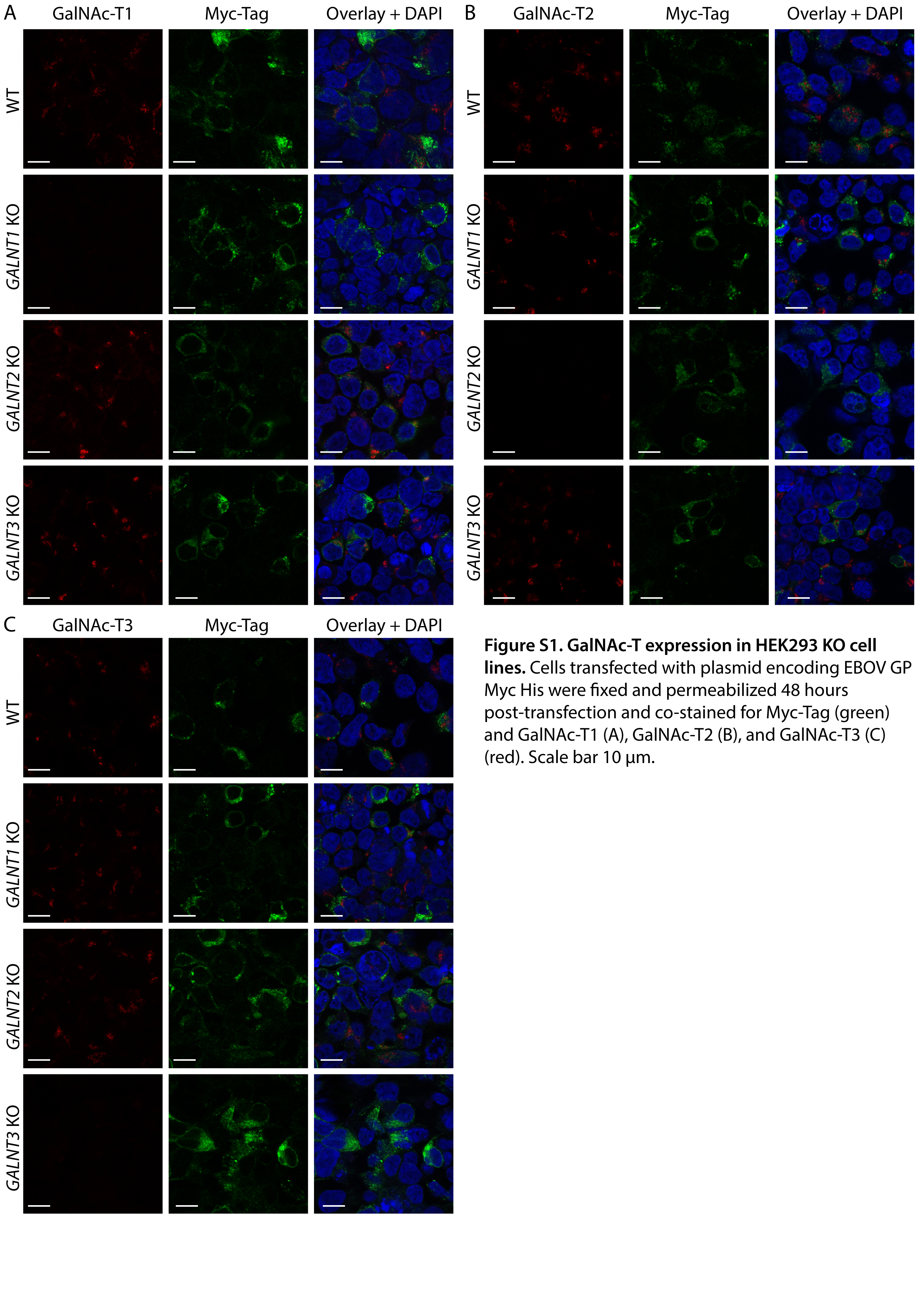

Supplement: Figure S1 — GalNAc-T expression in HEK293 KO cell lines. [file jvi.00524-24-s0002.jpg]

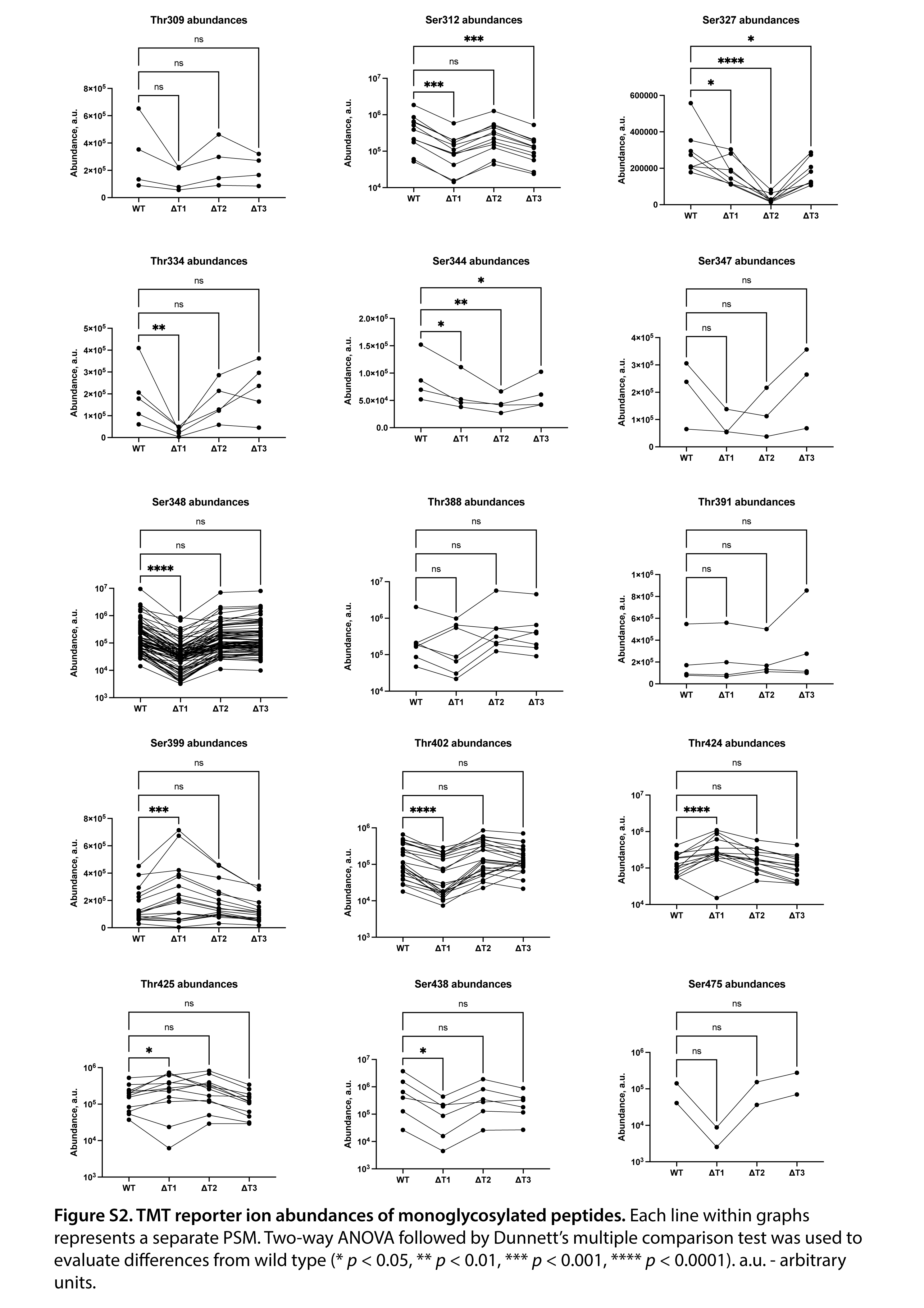

Supplement: Figure S2 — TMT reporter ion abundances of monoglycosylated peptides. [file jvi.00524-24-s0003.jpg]

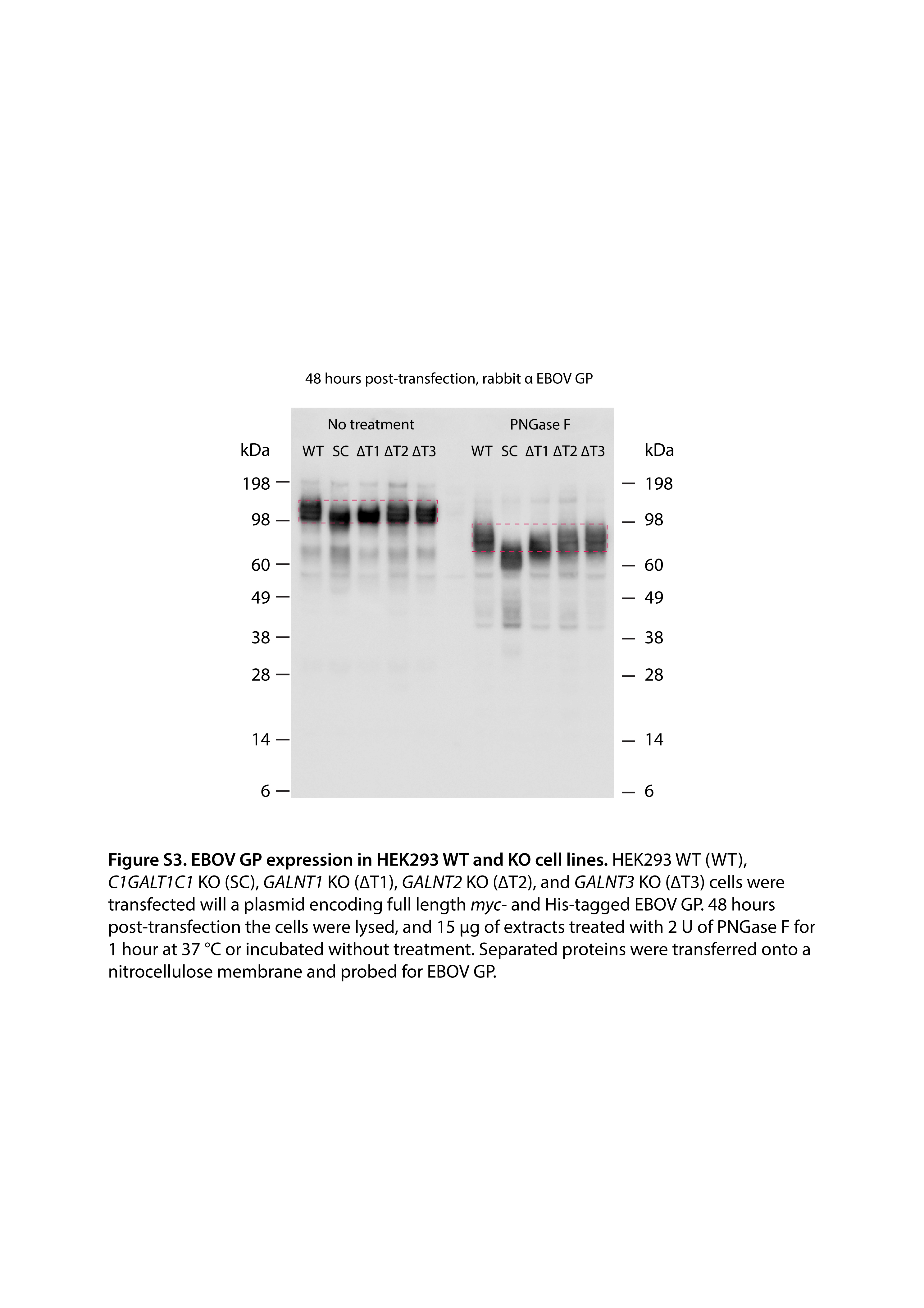

Supplement: Figure S3 — EBOV GP expression in HEK293 WT and KO cell lines. [file jvi.00524-24-s0004.jpg]

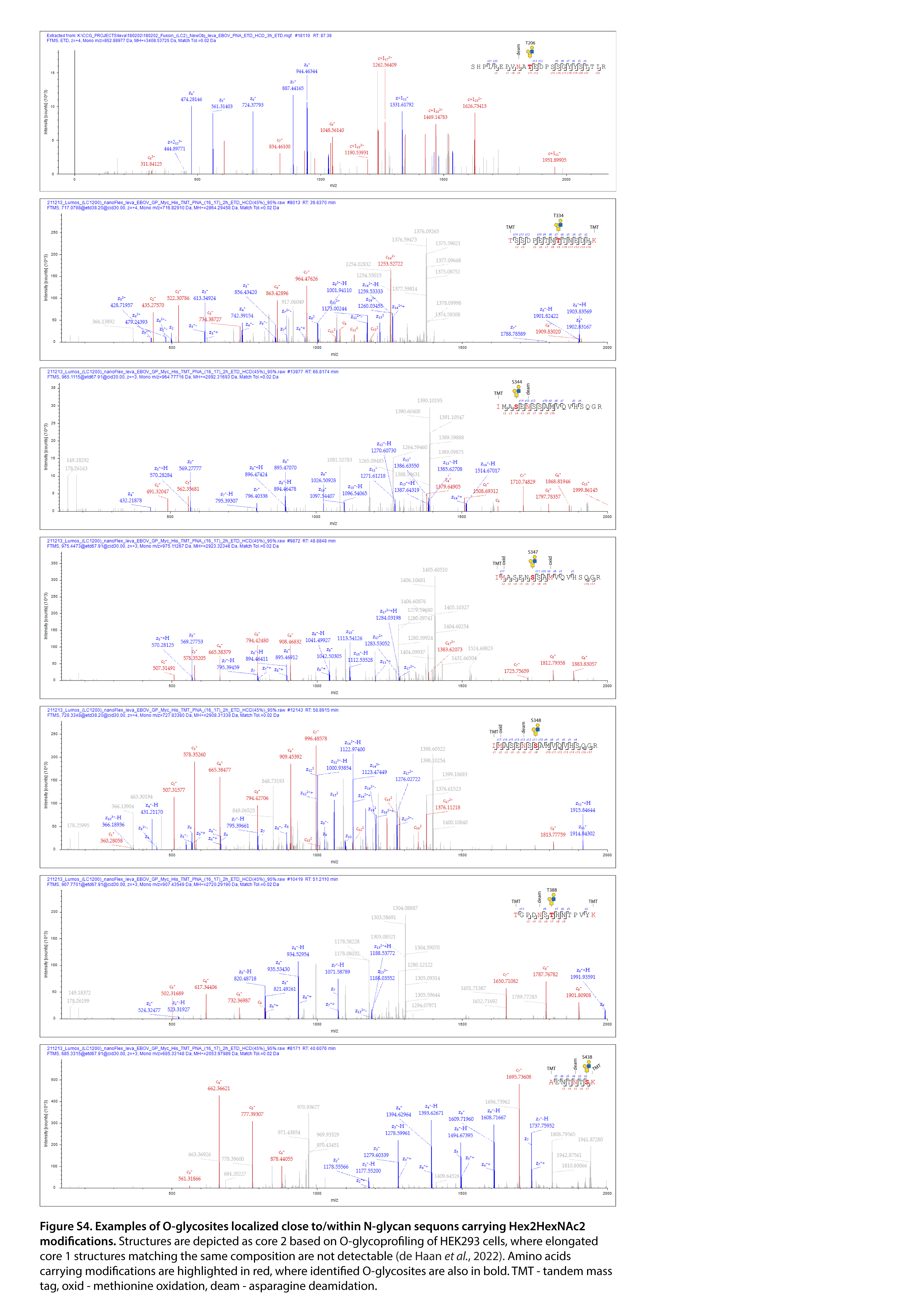

Supplement: Figure S4 — Examples of O-glycosites localized close to/within N-glycan sequons carrying Hex2HexNAc2 modifications. [file jvi.00524-24-s0005.jpg]

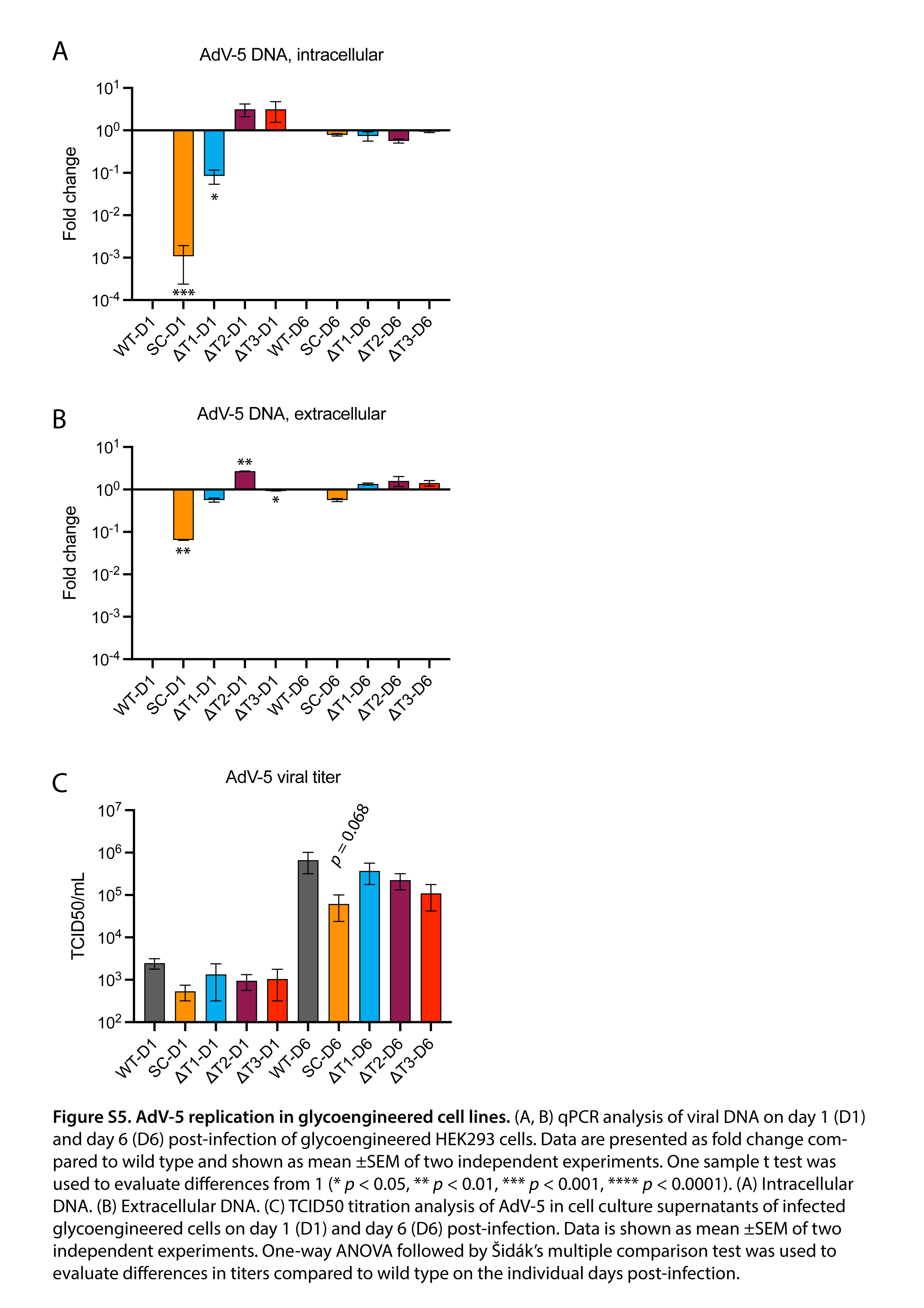

Supplement: Figure S5 — AdV-5 replication in glycoengineered cell lines. [file jvi.00524-24-s0006.jpg]
