# Supplementary material for: Targeting host O-linked glycan biosynthesis affects Ebola virus replication efficiency and reveals differential GalNAc-T acceptor site preferences on the Ebola virus glycoprotein
Source: J Virol. 2024 May 17;98(6):e00524-24. doi: 10.1128/jvi.00524-24 (PMC11237518; doi:10.1128/jvi.00524-24)
Supplement: Supplemental text — Legends for supplemental videos. [file jvi.00524-24-s0007.docx]

**Supplemental Text**

**Supplemental Video Legends**

**Video S1. MD simulation of O-glycosylated EBOV GP with isoform-specific glycosites highlighted.** A section of a molecular dynamics (MD) trajectory (covering a timescale of about 60 ns) is shown. The MLD is shaded in iceblue and the GCD is shaded in lime. GalNAc-T1, -T2, and -T3 regulated O-glycosites are highlighted in cyan, purple, and red, respectively. The remaining O-glycans are shown in white. Chain A contains VLP-derived glycosites, chain B – recombinant GP-derived glycosites, and chain C – combined maximum capacity. Putative N-glycans are shown in blue.

**Video S2.** **3D model of O-glycosylated EBOV GP with isoform-specific glycosites highlighted.** Last snapshot of the timescale covered in Video S1.

**Video S3.** **MD simulation of O-glycosylated EBOV GP with O-glycans colored by structure.** A section of a molecular dynamics (MD) trajectory (covering a timescale of about 60 ns) is shown. The MLD is shaded in iceblue and the GCD is shaded in lime. Identified O-glycans are colored based on the longest site-specific structure identified, as indicated in Fig 3D legend. Putative N-glycans are shown in blue.

**Video S4.** **3D model of O-glycosylated EBOV GP with O-glycans colored by structure.** Last snapshot of the timescale covered in Video S3.
